# Protein Structure Classification and Loop Modeling Using Multiple Ramachandran Distributions^*^

**DOI:** 10.1016/j.csbj.2017.01.011

**Published:** 2017-02-08

**Authors:** Seyed Morteza Najibi, Mehdi Maadooliat, Lan Zhou, Jianhua Z. Huang, Xin Gao

**Affiliations:** a Department of Statistics, College of Sciences, Shiraz University, Shiraz, Iran; b Department of Mathematics, Statistics and Computer Science, Marquette University, WI 53201-1881, USA; c Department of Statistics, Texas A&M University, TX 77843-3143, USA; d Computational Bioscience Research Center (CBRC), Computer, Electrical and Mathematical Sciences and Engineering Division, King Abdullah University of Science and Technology (KAUST), Thuwal 23955-6900, Saudi Arabia; e Center for Human Genetics, Marshfield Clinic Research Institute, Marshfield, WI 54449, USA

**Keywords:** Bivariate splines, Log-spline density estimation, Protein structure, Ramachandran distribution, Roughness penalty, Trigonometric B-spline, Protein classification, SCOP

## Abstract

Recently, the study of protein structures using angular representations has attracted much attention among structural biologists. The main challenge is how to efficiently model the continuous conformational space of the protein structures based on the differences and similarities between different Ramachandran plots. Despite the presence of statistical methods for modeling angular data of proteins, there is still a substantial need for more sophisticated and faster statistical tools to model the large-scale circular datasets. To address this need, we have developed a nonparametric method for collective estimation of multiple bivariate density functions for a collection of populations of protein backbone angles. The proposed method takes into account the circular nature of the angular data using trigonometric spline which is more efficient compared to existing methods. This collective density estimation approach is widely applicable when there is a need to estimate multiple density functions from different populations with common features. Moreover, the coefficients of adaptive basis expansion for the fitted densities provide a low-dimensional representation that is useful for visualization, clustering, and classification of the densities. The proposed method provides a novel and unique perspective to two important and challenging problems in protein structure research: structure-based protein classification and angular-sampling-based protein loop structure prediction.

## 1 Introduction

Proteins are large biomolecules or macromolecules that perform a vast array of functions for the biological processes within the cell of organisms. A protein is a linear chain of amino acids, each of which is composed of an amino group (–NH_2_), a central carbon atom (C_*α*_), a carboxyl group (–COOH), and a side-chain group that is attached to C_*α*_ and is specific to each amino acid. Depending on the amino acid sequence (different amino acids have different biochemical properties) and interactions with their environment, proteins fold into a three-dimensional structure, which allows them to interact with other proteins and molecules to perform their function. Hence, an important topic in the field of structural biology is the determination of the three-dimensional (3D) structure of a protein. In a protein, each amino acid is called a residue and the chain of carbon, nitrogen and oxygen atoms are referred to as the backbone. While the side-chain structures determine local structures and interactions of the amino acids of the protein, the backbone structure determines the overall shape of the protein and is the focus of much research.

The backbone conformation of proteins can be represented equivalently by Cartesian coordinates of carbon, nitrogen and oxygen atoms, or the backbone dihedral angles (*ϕ*,*ψ*), and *ω*, with the assumption of standard bond lengths and angles. Moreover, the global folds of proteins can be equivalently represented by either the Cartesian coordinates of *C*_*α*_ traces or the 2 pseudo-angles (*θ*,*τ*) between the two consecutive planes formed by 4 successive *C*_*α*_. The Ramachandran plot, a scatter plot of *ϕ* vs. *ψ*, can reflect the allowed regions of conformational space available to protein chains. By analogy to Ramachandran's concept of dihedral angles, the pseudo-Ramachandran plot, a scatter plot of *θ* vs. *τ*, can provide a distinctive classification of protein structures and largely contribute to different applications [1].

In the development of protein tools over the last two decades, the angular representation of proteins and Ramachandran plots have been applied in various protein structure-related problems, such as protein structural model checking [2], [3], [4], structure prediction [5], [6], [7], [8], [9], model quality assessment [10], [11], [12], prediction server ranking [13], [14], protein structure alignment [15], [16], free energy function learning [17], [18], [19], molecular dynamics simulation [20], empirical energy functions [21] and classification functions such as backbone-dependent rotamer library [22], [23].

Since the seminal work of Ramachandran et al. [24], the two-dimensional histogram of Ramachandran plot has been commonly used to determine accessible regions and validate new protein structures [2], [3]. The histogram is a rough non-parametric density estimation where the number of parameters is equal to the number of data points. Furthermore, because of the circular nature of the protein angles, the traditional parametric or non-parametric density estimation methods cannot be used for estimating Ramachandran distributions. In the last decade, novel parametric and non-parametric methods have been introduced to address this problem. The parametric methods propose to use directional distributions such as von Mises distribution or short Fourier series that are naturally designed for periodic data [25], [26], [27], [28], [29]. On the other hand, the non-parametric techniques use kernel density estimates with periodic kernels, Dirichlet process with boundary modification, or a mixture of directional distributions [30], [31], [32].

Depending on the purpose of the study, one may produce Ramachandran plots based on residues associated with some specific amino acids, and/or some specific structural elements. In some cases, the number of residues (data points) is too small, and that makes it challenging to obtain reliable bivariate densities using techniques that estimate each Ramachandran distribution separately. An intuitive solution to this problem is to borrow information from a group of Ramachandran plots that has some common features. To this end, Lennox et al. [33] proposed a hierarchical Dirichlet process technique based on bivariate von Mises distributions that can simultaneously model angle pairs at multiple sequence positions. This method is typically used for predicting highly variable loop and turn regions. Ting et al. [34] and Joo et al. [35] also used this technique with further modification to produce near-native loop structures. In another approach, Maadooliat et al. [36] proposed a penalized spline collective density estimator (PSCDE) to represent the log-densities based on some shared basis functions. This method showed some significant improvements for loop modeling of the hard cases in a benchmark dataset where existing methods do not work well [36].

Comparing to other competitive approaches, PSCDE is more efficient in estimating the densities in the sparse regions by incorporating the shared information among the distributions. In this technique, the bivariate log-densities are represented using a common set of basis functions. Each log-density has its own coefficient vector in the basis expansion, and it can be used for clustering and classification of the densities. Furthermore, using a common set of basis functions significantly reduces the number of parameters to be estimated. This method has been applied to estimate the neighbor-dependent Ramachandran distributions to make the angular-sampling-based protein structure prediction more accurate. In this paper, we make an innovative and constructive development over the PSCDE method.

The PSCDE method is constructed based on Bernstein-Bézier spline basis functions defined over triangles to estimate the log-densities in a complex domain [36]. In simple words, in PSCDE,we artificially extended the constraints of the adjacent triangles to the triangles in boundaries in order to estimate the densities in a two-dimensional circular domain. Here, we propose an alternative approach that uses the tensor product of trigonometric B-spline basis to handle the angular nature of the data. The main advantage of the proposed method is that there is no need to implement any further constraints to take into account the continuity and circularity of the data since the new bases are trigonometric functions that are smooth and intrinsically periodic. Another improvement in the proposed procedure is on selecting the smoothing parameter. In the existing PSCDE procedure, the tuning parameter is selected using the Akaike Information Criterion. Therefore a grid search is needed to choose the optimal tuning parameter and that could become time-consuming, especially if different tuning parameters are used for different basis functions. Following Schellhase and Kauermann [37], we propose to update the smoothing parameter within the Newton–Raphson iterative procedure that is used for the density estimation.

The PSCDE method is originally applied to the protein loop modeling problem. Here, we focus on a new application and use an extension of PSCDE to the protein structure classification problem. There is a large literature on the classification of the protein structures in the Protein Data Bank (PDB) [38], [39], [40]; because a good classification can reveal the evolutionary relationship between the proteins and step toward understanding the protein functions. While a vast majority of the literature deals with the protein classification in a pairwise structural comparison framework, the proposed estimated densities can be used as an alternative technique based on angular representation for the structural classification.

Specifically, the estimated angular density corresponding to a protein structure has a basis expansion whose coefficients can be used as an input to a clustering algorithm. Furthermore, most of the existing techniques for protein classification are using sequence and/or 3D structure comparison to classify the proteins based on some (dis)similarity scores obtained after pairwise alignments. The proposed method is an alignment-free procedure that provides a vector of coefficients (i.e. features), associated with each structure (density), that can be directly used to classify the proteins.

We also applied the proposed method to the loop modeling problem and compared the result with the other methods in the online supplementary. In this application, we trained the neighbor-dependent distributions of the backbone dihedral angles (i.e., neighbor-dependent Ramachandran distributions) using the new collective density estimation approach and fed the results into the Rosetta loop modeling procedure to study the accuracy and efficiency of the Rosetta server in predicting the loop regions. The main concern of using the neighbor-dependent Ramachandran distributions is that we are partitioning the data into smaller groups, some partitions may end up with a limited number of observations, and therefore we may lose accuracy in estimating the Ramachandran distributions due to the data sparsity. The proposed collective estimation procedure can overcome this difficulty and thereby improve the accuracy of the estimated densities. We encourage the interested readers to read the online supplementary materials for the implementation of the proposed method on loop-modeling application.

The rest of the paper is organized as follows. Section 2 introduces the penalized spline collectively density estimator procedure based on the new trigonometric basis functions to incorporate the circular nature of data. Section 3 presents the protein structure classification problem and the implementation of the new procedure for this application. Section 4 concludes the paper with a discussion. A web-based toolbox is also introduced in the Appendix to illustrate the advantages of the proposed technique. This toolbox can be used further by the research community to obtain the collective estimation of Ramachandran distributions for any other related application (e.g. backbone-dependent rotamer library [22], [23]).

## 2 Collective Estimation of Multiple Probability Density Functions

In this section, we review and extend a procedure for estimating the multiple probability density functions, known as the PSCDE [36]. Suppose that we observe data from *m* bivariate probability distributions with the density functions *f*_*i*_, *i* = 1,⋯ ,*m*. We assume that each log-density can be represented by a set of common basis functions. Therefore we write each log-density function as

(1) $$\log\left\{f_{i}(x)\right\}=\omega_{i}(x)+c_{i},$$

where *ω*_*i*_(***x***) is a linear combination of the basis functions {*ϕ*_*k*_,*k* = 1,…,*K*} such that

(2) $$\omega_{i}(x)=\overset{K}{\underset{k=1}{\sum }}\phi_{k}(x)\alpha_{ik}\;\forall i=1,\ldots ,m,$$

and *c*_*i*_ is a normalizing constant $\left(c_{i}=-\log\int \exp\omega_{i}(x)dx\right)$ to ensure that each *f*_*i*_ is a valid density function. In our setting, the value of *K* and the basis functions (*ϕ*_*k*_’s) are not pre-specified and will be determined based on data. We assume that *ϕ*_*k*_’s fall in a low-dimensional subspace of a function space spanned by a rich family of fixed basis functions, {*b*_*ℓ*_(***x***),*ℓ* = 1,…,*L*},(*L* ≫ *K*), such that

$$\phi_{k}(x)=\overset{L}{\underset{\ell =1}{\sum }}b_{\ell }(x)\theta_{\ell k}.$$

**Fig. 1:**
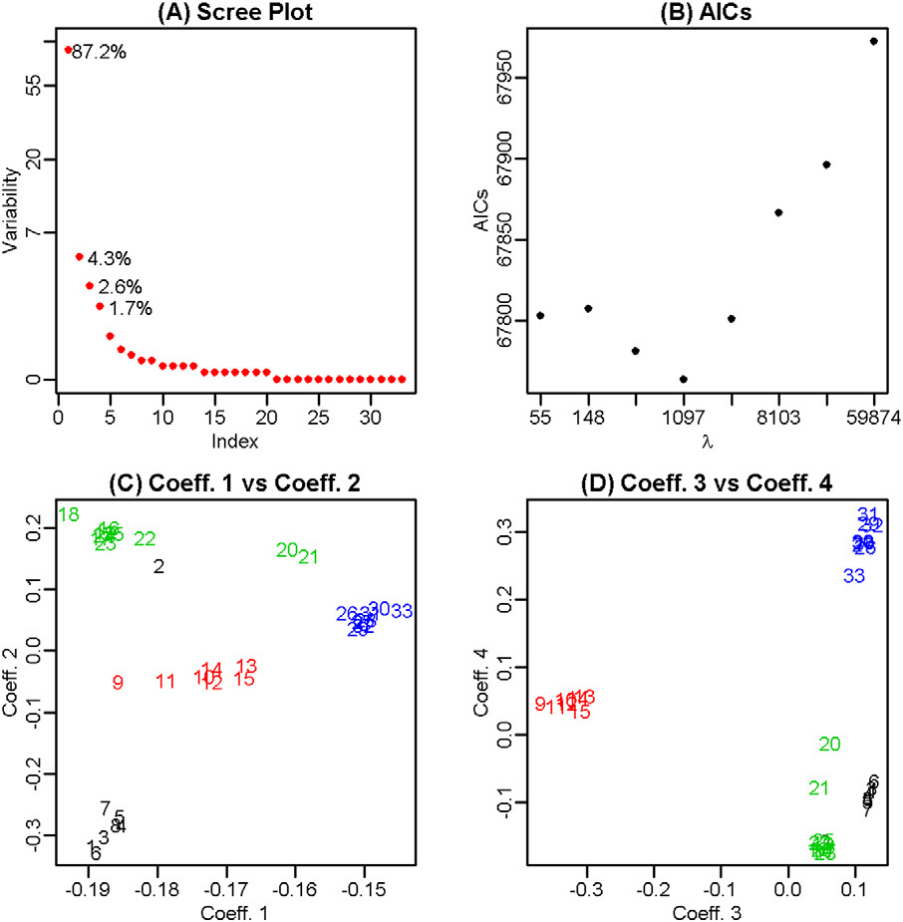
A classification task with 33 domains from four *Species* of the same protein class, separated at the bottom of SCOP hierarchy with PSCDE approach [36]. (A) The scree plot with numbers showing the percentage of variability explained by the leading components; (B) the AIC plot; (C) the scatter plot of coefficients 1 vs 2; and (D) the scatter plot of coefficients 3 vs 4.

**Fig. 2:**
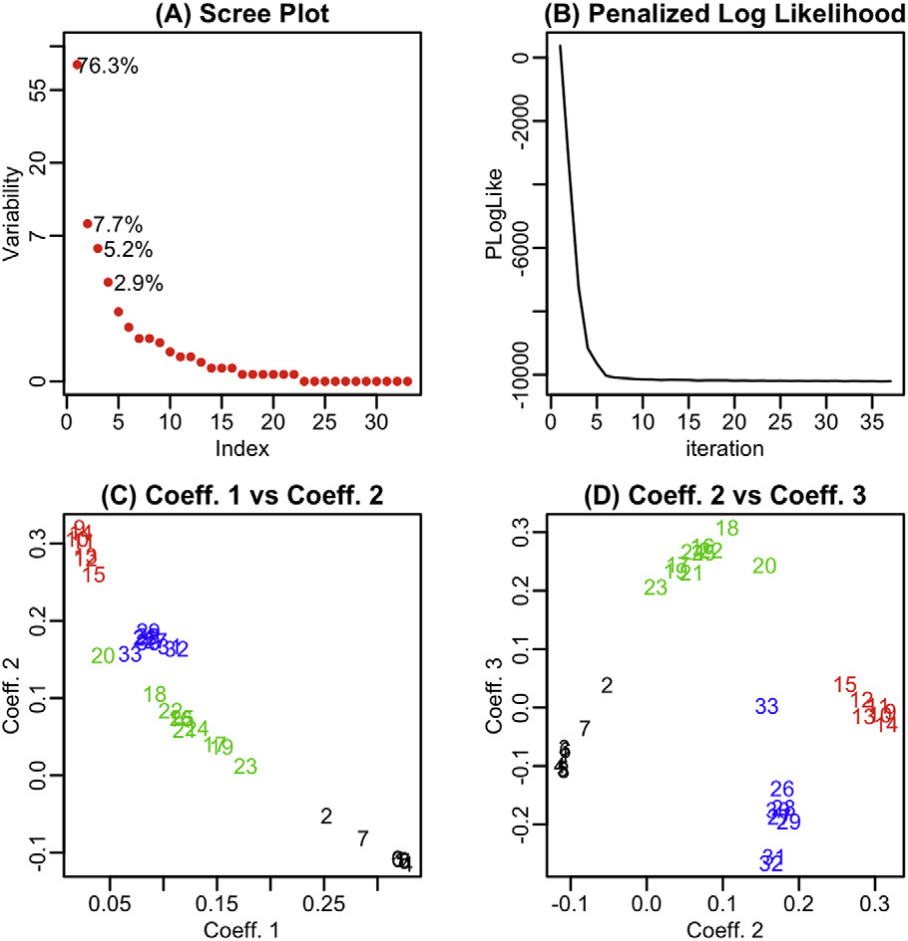
A classification task with 33 domains from four *Species* of the same protein class, separated at the bottom of SCOP hierarchy with PSCDE(T) approach. (A) The scree plot with numbers showing the percentage of variability explained by the leading components; (B) the trace of the penalized log-likelihood function; (C) the scatter plot of coefficients 1 vs 2; and (D) the scatter plot of coefficients 2 vs 3.

This framework provides a common set of basis functions to represent the log-densities. Also, each density in this model is represented with a set of coefficients *α*_*ik*_, *k* = 1,…,*K*, which can be used as an excellent feature for comparison, assessment and classification of the densities. Furthermore, similar to the scree plot in principal component analysis (PCA), one may plot the sum of square of the component coefficients $\left(g(k)=\sum_{i}\alpha_{ik}^{2}\right)$ as a function of component index, to select number of significant components, *K*, e.g. see Figs. 1A and 2A.

Here, we use the tensor product technique to construct bivariate trigonometric splines that are smooth and intrinsically periodic in one or two directions. The details on how to construct the basis functions are given in Section 2.1. To further simplify the presentation, let ***ϕ***(***x***) = (*ϕ*_1_(***x***),*ϕ*_2_(***x***),…,*ϕ*_*K*_(***x***))^⊤^, ***α***_*i*_ = (*α*_*i*1_,*α*_*i*2_,…,*α*_*iK*_)^⊤^, **b**(***x***) = (*b*_1_(***x***),*b*_2_(***x***),…,*b*_*L*_(***x***))^⊤^, ***θ***_*k*_ = (*θ*_1*k*_,*θ*_2*k*_,…,*θ*_*Lk*_)^T^ and **Θ** = (***θ***_1_,***θ***_2_,…,***θ***_*K*_), then *ω*_*i*_(***x***) given in Eq. (2) can be written as

(3) $$\omega_{i}(x)=\phi {\left(x\right)}^{⊤}\alpha_{i}=b{\left(x\right)}^{⊤}\Theta \alpha_{i},\;i=1,\ldots ,m.$$

If we evaluate the densities on common regular grids (***x***_*j*_, *j* = 1,⋯ ,*n*) in the circular plane, we may further simplify the presentation of the densities in an *n* × *m* matrix: **Ω** ={*ω*_*i*_(***x***_*j*_)}^⊤^. Specifically, let **B** = (**b**(***x***_1_),**b**(***x***_2_),…,**b**(***x***_*n*_))^⊤^, and ***A*** = (***α***_1_,***α***_2_,…,***α***_*m*_)^⊤^, then Eq. (3) can be written in the matrix form, **Ω** = **BΘA^T^**, where the parameters to be estimated are (**Θ**,**A**). To address the identifiability issue raised by the product of two matrices (**Θ**,***A***), we follow the remedy given in [36] based on the singular value decomposition (SVD) technique.

Now, by assuming observations *x*_*ij*_, *j* = 1,…,*n*_*i*_ from the *i*th group, i=1,…,m, the log-likelihood function has the following form:

(4) $$\ell (\Theta ,A)=\overset{m}{\underset{i=1}{\sum }}\overset{n_{i}}{\underset{j=1}{\sum }}\left\{\omega_{i}\left(x_{ij}\right)+c_{i}\right\}.$$

To obtain smooth densities, the parameters can be estimated by introducing the roughness penalty [41] and minimizing the penalized likelihood criterion:

(5) $$-2\;\ell (\Theta ,A)+\lambda \;\text{trace}(\Theta^{⊤}D\Theta ),$$

where ***D*** penalizes wiggliness (induces smoothness) and *λ* > 0 is the tuning parameter. We then use the alternating blockwise Newton–Raphson algorithm in Maadooliat et al. [36] to minimize the penalized likelihood function.

There are different well-known methods to select the tuning parameter. A commonly used technique is to choose the tuning parameter, *λ*, that minimizes the Akaike Information Criterion (AIC) [42]:

AIC(λ)=−2ℓ(Θ,A)+2df(λ),

where *ℓ*(**Θ**,***A***) is the log likelihood function and df(*λ*) is the degrees of freedom, defined as:

$$\text{df}(\lambda )=\overset{K}{\underset{k=1}{\sum }}\text{trace}\left\{{\left[\frac{\partial^{2}\ell (\Theta ,A)}{\partial \theta_{k}\partial \theta_{k}^{⊤}}+\lambda D\right]}^{-1}\left[\frac{\partial^{2}\ell (\Theta ,A)}{\partial \theta_{k}\partial \theta_{k}^{⊤}}\right]\right\}.$$

Selecting the tuning parameter that minimizes the AIC, requires training the model for different values of *λ*’s and then pick the one that minimizes the criterion function, which can be very expensive in time. Instead, we present an alternative procedure that updates the value of the tuning parameter within the Newton–Raphson iterations. This idea has been used in generalized mixture model to iteratively update the smoothing parameter [43]. Schellhase and Kauermann [37] extended this approach for density estimation. We borrow their formulation, and use the parameter estimates in the *i*th step to update the tuning parameter, ${\hat{\lambda }}_{i+1}$, through

(6) $${\hat{\lambda }}_{i+1}^{-1}=\frac{\text{trace}\left({\hat{\Theta }}_{i}^{⊤}D{\hat{\Theta }}_{i}\right)}{\text{df}\left({\hat{\lambda }}_{i}\right)-(a-1)},$$

where *a* is the order of the differences used in the penalty matrix ***D*** (see Section 2.1). From what we have seen in the implementation of the new procedure, updating the tuning parameter within the Newton–Raphson iterations, on average, does not increase the number of the iterations required to converge. Therefore the new procedure obtains the final result *p* times faster than the older procedure, where *p* is the number of *λ*’s used in the grid search to minimize the AIC.

In the following subsection, we obtain the trigonometric basis functions and the penalty matrix that has been used in minimizing the penalized likelihood function (Eq. (5)).

### 2.1 Basis Functions and the Penalty Matrix

There are a variety of basis functions that can come in handy depend on the dimensionality of the problem and the data structure. In this context, the circular nature of the protein angles is an obstacle that prevents us from using the standard B-spline functions. Maadooliat et al. [36] proposed to use bivariate spline functions over triangulations, and they artificially extended the constraints for two adjacent triangles [44] to the triangles in boundaries. Triangulation is a sophisticated procedure that works perfectly for complex geometries with unbalanced observations over irregular grid points. For Ramachandran plot, we evaluate the densities over regular grid points in a smooth rectangular plane that is obtained by unfolding a simple manifold (torus or sphere), and it is better if we can avoid such sophisticated procedure. Furthermore, extending the triangulation technique beyond the bivariate case, and implementing the PSCDE via triangulations in higher dimensions is not straightforward.

A frequently used basis functions for Euclidean space is the tensor product of standard B-spline functions which is appealing and very easy to use in the real world applications [45]. With some small alteration, the tensor product of trigonometric spline can be defined by sin and cos functions which are smooth and naturally periodic functions [46]. Moreover, this method can be easily applied to higher dimensional density estimation.

We need to develop rich set of basis functions {*b*_*ℓ*_(***X***),*ℓ* = 1,…,*L*}, that is required for estimating the Ramachandran or pseudo-Ramachandran distributions, over the support set (Ω or Ω*′*) which can be defined as

(7) $$\Omega =\{-\pi \leq \phi \leq \pi \;\text{and}\;-\pi \leq \psi \leq \pi \}\;\text{or}\;\Omega^{\prime }=\{-\pi \leq \theta \leq \pi \;\text{and}\;0\leq \tau \leq \pi \}.$$

From a geometric point of view, Ω resembles the surface of a torus with some fixed minor/major radiuses and Ω*′* represents the surface of a sphere with fixed radius. In fact, the existing parametric models take into account the topology and develop a parametric framework on surfaces of a torus or sphere with some fixed radiuses to model the bivariate densities [32], [47], [48]. In contrast, non-parametric methods use either a periodic kernel or some boundary modification technique to address this issue.

Here we present the tensor product of two sets of trigonometric basis functions and construct the bivariate bases that can be used to represent the space for two dihedral angles (*ϕ*,*ψ*) defined over Ω. One may proceed with a similar procedure based on the Kronecker product of a trigonometric spline and a standard B-spline to obtain the bivariate basis representation for the pair of dihedral, planar angles (*θ*,*τ*) defined over Ω*′*.

A univariate normalized trigonometric spline with *κ* knots, (*x*_1_,*x*_2_,⋯ ,*x*_*κ*_), and order of *ν*, can be represented recursively as a periodic spline on a circle; see Schumaker [49, ch. 8] for details. In specific, for every *ϕ* within the interval [*x*_*i*_,*x*_*i* +*ν*_] the spline functions are defined as

(8) $$\begin{array}{c} S_{i}^{1}(\phi )=\left\{\begin{array}{ll} 1\; & x_{i}\leq \phi \leq x_{i+1}\\ 0\; & \text{o.w.} \end{array}\right),\\ S_{i}^{\nu }(\phi )=\frac{\sin\left(\frac{\phi -x_{i}}{2}\right)}{\sin\left(\frac{x_{i+\nu -1}-x_{i}}{2}\right)}S_{i}^{\nu -1}(\phi )+\frac{\sin\left(\frac{x_{i+\nu }-\phi }{2}\right)}{\sin\left(\frac{x_{i+\nu }-x_{j+1}}{2}\right)}S_{i+1}^{\nu -1}(\phi ). \end{array}$$

The same methodology should be used to create basis functions for dihedral angle, *ψ*. The main advantage of using these linearly independent basis functions over the standard B-spline choice is that the continuity of the tangent plane for any smooth function on surface of a sphere is the result of the former one. Therefore, there is no need to introduce any periodic constraints for the trigonometric spline functions (for more details see Schumaker and Traas [50]), due to the fact that each piece lies in $span(F_{m})$, where:

$$F_{m}=\left\{\begin{array}{ll} \left\{\cos(\phi /2),\sin(\phi /2),\ldots ,\cos((2q-1)\phi /2),\sin((2q-1)\phi /2)\right\}\; & if\;\nu =2q,\\ \{\cos(\phi ),\sin(\phi ),\ldots ,\cos(q\phi ),\sin(q\phi )\}\; & if\;\nu =2q-1. \end{array}\right)$$

In matrix form, we denote ***B***_*ϕ*_ and ***B***_*ψ*_ to be the matrices that represent the trigonometric basis functions associated to *ϕ* and *ψ* directions with ranks *M* and *N* respectively. The matrix ***B*** that represents the bivariate spline basis functions can be then obtained from the Kronecker product of ***B***_*ϕ*_ and ***B***_*ψ*_:

$$B=B_{\phi }⊗B_{\psi },$$

where the symbol ⊗ is used to represent the Kronecker product.

It should be noted that the number of knots, *κ*, directly influence the smoothness of the estimated functions. The smaller *κ* results smooth, but biased estimates. While increasing *κ* will reduce the bias, but it will consequently increase the variability and therefore, we end up with some rough estimates. It is customary to have a large number of knots in the model and control the smoothness of estimates by introducing a roughness penalty into the likelihood function, to control the bias-variance tradeoff. Here, we monitor the roughness of the estimated functions by using difference penalty [51] to achieve the appropriate level of smoothness. In a nutshell, the variability is controlled through a difference function of order *a*, Δ_*a*_, where Δ_1_***θ***_*k*_ : =***θ***_*k*_ −***θ***_*k* −1_, and Δ_*a*_ is obtained recursively. For example, the second order difference function, Δ_2_, has the following form:

$$\Delta_{2}\theta_{k}:=\Delta_{1}\Delta_{1}\theta_{k}=\theta_{k}-2\theta_{k-1}+\theta_{k-2}.$$

We may write the difference functions Δ_*a*_ into a matrix form, ***L***_*a*_. For example, for *a* = 1 we have

$$L_{1}={\left[\begin{array}{lllll} 1 & -1 & 0 & \ldots & 0\\ 0 & 1 & -1 & \ddots & 0\\ \vdots & \ddots & \ddots & \ddots & 0\\ 0 & \cdots & 0 & 1 & -1 \end{array}\right]}_{\left(M-1\times M\right)}$$

The positive definite penalty matrix used to control the smoothness in the *ϕ* direction is defined as *D*^*ϕ*^, and it has the following quadratic form: $D^{\phi }=L_{a}^{⊤}L_{a}$. Now, we may use the tensor product technique to derive the penalty matrix for the bivariate domain, (*ϕ*,*ψ*), as the following:

(9) $$D=\left[I_{N}⊗D^{\phi }+D^{\psi }⊗I_{M}\right],$$

where $D^{\phi }={\left(L_{a}^{\phi }\right)}^{⊤}L_{a}^{\phi }$ and $D^{\psi }={\left(L_{a}^{\psi }\right)}^{⊤}L_{a}^{\psi }$.

We now have the required tools to proceed with the estimation procedure. The minimization of the penalized likelihood function (Eq. (5)) can be obtained through the Newton–Raphson algorithm, of which the details can be found in [36]. After convergence, the densities can be obtained using Eq. (1). From now on, we refer to our new procedure that uses the trigonometric basis expansion in PSCDE as PSCDE(T).

## 3 Application: Protein Structure Classification

In this section, we introduce an application of collective density estimation in protein structural comparison. To evaluate the proposed method, we designed four protein clustering tasks from the Structural Classification of Proteins (SCOP) database, and then try to cluster the proteins in each task without knowing their labels in the SCOP tree. The final clustering result of PSCDE(T) is compared with seven competitive approaches using two external measures (the descriptions are given in 3.3, 3.4), where SCOP labels are used as the gold standard. Since the class labels were not used, this is a clustering or unsupervised learning problem.

### 3.1 Structural Classification of Proteins

The Structural Classification of Proteins is a widely used database that stores the results of classification of known protein structures and is available at http://scop.mrc-lmb.cam.ac.uk/scop/. The SCOP has been constructed manually by visual inspection and comparison of structures. Since manual inspection and classification is time-consuming and subjective, automated classification methods have been developed in the past two decades, including alignment-based methods [52], [53], [54], alignment-free methods [55], and consensus methods [56], [57]. However, it is well acknowledged that a reliable automatic protein classification method is not yet available, partly due to the fact that most of the existing methods depend on distance-based similarity measures and are biased by sequence alignments [55], [58]. In this section, we report the results from some experiments of using the SCOP database as a benchmark to evaluate the potential use of angular distributions for automatic protein structure classification. In contrast to the existing protein structure classification methods, our method is completely alignment-free and does not depend on sequence similarity or distance-based measures, thus provides a unique perspective to the problem.

In the SCOP database, protein domains are classified hierarchically according to their sequential, structural and functional relationship. From top to bottom, the SCOP hierarchy comprises the following seven levels: *Class*, *Fold*, *Superfamily*, *Family*, *Protein*, *Species*, and *Domain*. The *Domain* level lists the individual protein domains of known structures. We refer to Murzin et al. [38] and Andreeva et al. [40] for more details regarding the description of the SCOP hierarchy and how the database is organized.

### 3.2 Task Designs

To evaluate the performance of PSCDE(T) in different datasets, we designed four SCOP tasks with “Easy”, “Somewhat Hard”, “Hard” and “Challenging” level of difficulty, that we call them SCOP.1 to SCOP.4, respectively:

1. SCOP.1 (Easy Task): In this task, we considered an easy protein classification. The goal is to classify 63 protein domains that were randomly selected from three remote Protein *Classes* in SCOP. The constituents of the collection of protein domains and the details of this SCOP tree are available in the online supplementary materials.
2. SCOP.2 (Somewhat Hard Task): We considered a protein classification task for which 33 domains were extracted from four *Species* under the same *Protein* subclass that belongs to the “all-alpha protein” *Class*. The constituents of the collection of domains and the details of the SCOP tree involving these domains are available in the online supplementary materials. This classification task is considered somewhat harder than the easy task, because the domains are very similar both sequentially and structurally—they are very close in the SCOP tree and depart only at the bottom (i.e., the *Species* level) of the SCOP hierarchy.
3. SCOP.3 (Hard Task): We considered a protein classification task for which 40 protein chains were randomly selected from three different *Fold*/*Superfamily* levels, where all chains belong to the “Alpha and beta proteins (a+b)” *Class*. The constituents of the collection of domains and the details of the SCOP tree involving these domains are available in the online supplementary materials. This classification task is considered harder than the SCOP.2, because the similarities within a group of chains branched out from a specific *Superfamily* level is not as strong as branching out at a specific *Species* levels. This task can be used to evaluate different methods in detecting the remote homology relationship at the *Superfamily* level.
4. SCOP.4 (Challenging Task): Fischer et al. [59] provided a challenging benchmark to assess the performance of a fold recognition method in an objective, unbiased and thorough way. We have selected 26 protein chains from their benchmark in the “All beta proteins” *Class* within three different *Folds*. This classification task is considered the hardest task in this paper, which is also indicated in [59].

After choosing the protein domains from the SCOP database, the complete information of the proteins were obtained from the Protein Data Bank (PDB). The PDB record of each protein structure contains its 3D atomic coordinates, secondary structure assignments, as well as atomic connectivity. While different types of dihedral/planar angles can be obtained using the atomic coordinates, we used the R package PRESS [60] to derive the (*θ*,*τ*) angles from the PDB files for each task. We observed that *θ* angles are within the range (75,165) and *τ*’s are within ( −180,180).

### 3.3 Protein Classification Approaches and Distance Matrices

Due to the tree based structure of the SCOP database, we use the agglomerative hierarchical clustering technique to group the protein structures. In order to do this, we need to feed in a pairwise (dis)similarity matrix as an input to the clustering algorithm. In this subsection, we illustrate how to obtain such (dis)similarity matrices to compare five non-density based and three density based approaches, respectively.

Since clustering cannot be directly performed on 3D protein structures, a protein structure or sequence comparison algorithm is usually applied to generate (dis)similarity scores between any pair of structures and such scores are then used for clustering [61]. We considered five such algorithms that cover a broad spectrum of existing methods:

• Needleman–Wunsch (NW) algorithm for global sequence alignment [62], with implementation available in the R package Biostrings;
• Smith–Waterman (SW) algorithm for local sequence alignment [63], with implementation available in the R package Biostrings;
• TM-align [64], available at http://zhanglab.ccmb.med.umich.edu/TM-align/;
• Yakusa [65], available at http://bioserv.rpbs.jussieu.fr/Yakusa/download/index.html;
• Dali [66], available at http://ekhidna.biocenter.helsinki.fi/dali_lite/downloads/v3/.

The first two methods are based on sequence comparison, and the other three methods are based on structure comparison. After we apply these five algorithms, we follow Sam et al. [61] to transform the similarity matrices to distance matrices.

We also considered three density based approaches: Kernel Density Estimator (KDE), PSCDE and PSCDE(T) for protein classification. We used Symmetric Kullback–Leibler Divergence (SKLD) between Ramachandran distributions to obtain pairwise distance matrices between proteins [14]. In the KDE, we used Gaussian kernel density estimation with slight modification to consider the angular structure of the data to obtain an estimate of each density separately [14].

In the PSCDE(T) method, we initialized the algorithm with the cubic B-spline basis functions with 5 degrees of freedom in the *θ* direction and the cubic trigonometric B-spline basis functions with 15 degrees of freedom in the *τ* direction. The final tensor product basis functions are obtained and evaluated over 90 gird points in each direction. Furthermore, we selected the number of common basis to be equal to the number of classes in the gold standard associated to each task (four common basis for SCOP.2, and three common basis for the remaining three tasks). In general, one may use scree plot based on the initial estimates (obtained by mapping the kernel density estimators to the column space of the basis expansion) or other approaches available in the literature to select the number of common basis. After estimating the parameters (**A**,**Θ**) using the Newton–Raphson algorithm, the densities can be obtained using Eq. (1). The PSCDE results can be obtained similarly. In order to have comparable initial basis functions for PSCDE, we partitioned the (*θ*,*τ*) domain to 64 similar right triangles with cubic bivariate B-spline basis functions over each triangle (see [36] for more details).

**Fig. 3:**
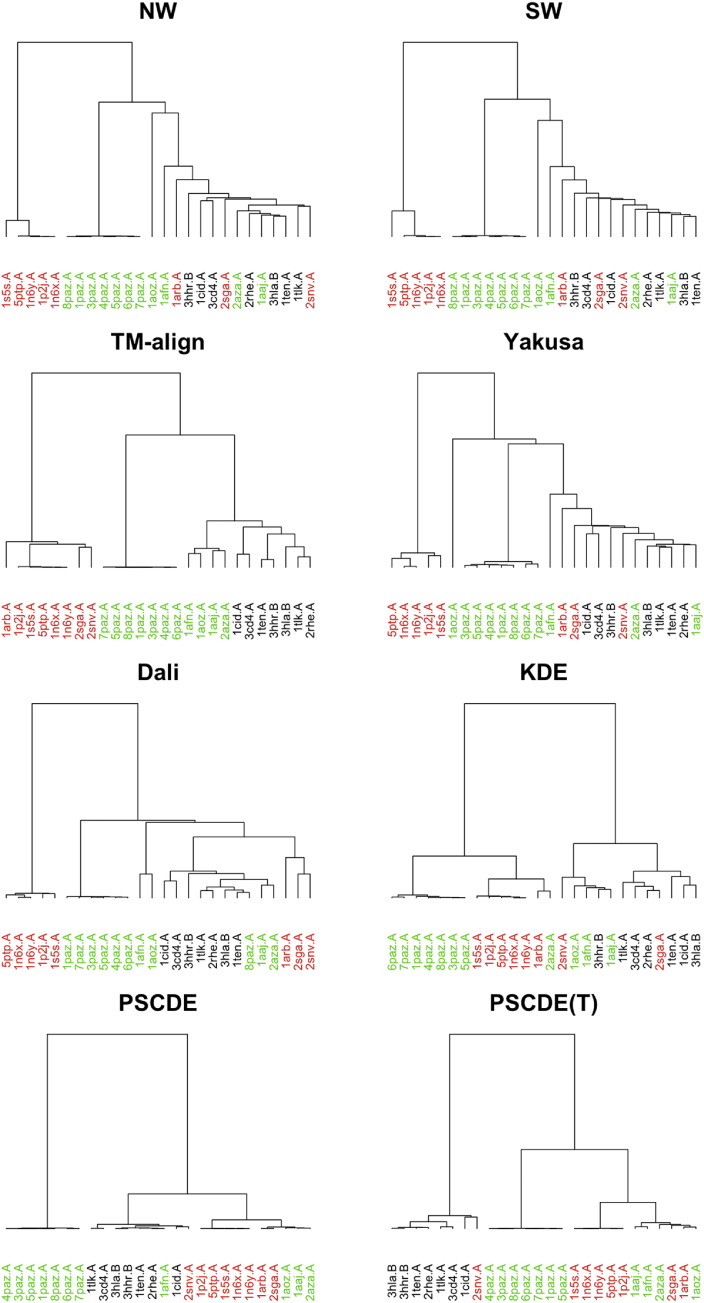
Dendrograms from hierarchical clustering for SCOP.4 task.

The distance matrices obtained for the above eight approaches: NW, SW, TM-align, Yakusa, Dali, KDE, PSCDE and PSCDE(T) are used as an input to the hierarchical clustering algorithm, implemented in the hclust function with option {method=”ward.D”} in the R package stats to obtain dendrograms [67] (e.g. see Fig. 3). In order to obtain the clusters, we cut the dendrograms of all eight approaches into the number of the original clusters in the SCOP database. To evaluate the performance of the proposed method in discovering the correct label (gold standard), we used two external measures that are commonly used in the clustering evaluation literature and discussed in Section 3.4.

### 3.4 External Evaluation Measures

Consider *A* and *B* be two clusterings of a dataset consisting of *N* records. Let *A* cluster the data in *r* clusters and define *a*_*i*_ as the size of cluster *i* = 1,…,*r*, and let *B* cluster the data in *c* clusters of size *b*_*j*_ for each cluster *j* = 1,…,*c* (Note that, in our comparison *r* = *c*). Given that *A* and *B* are partitions of the same data it is possible to count the elements that belong both to cluster *i* and *j*. Let *n*_*ij*_ denote the number of records shared between cluster *i* and *j*. The overlap between two clusterings can be represented in matrix form by a *r* × *c* contingency table *M* such as the one in Table 1. We refer to $a_{i}=\sum_{j}n_{ij}$ as the row marginals and to $b_{j}=\sum_{i}n_{ij}$ as the column marginals.

Here, we have used two external measures as follows:

1. Normalized Mutual Information (NMI): In the information theory, the mutual information of two random variables is a measure of the mutual dependence between the two variables. The concept of mutual information is intricately linked to that of entropy of a random variable. The entropy in clustering is defined as the expected value of its information content if it is seen as a random variable. We can therefore define entropy for clustering A and B as$H(A)=-\sum_{i=1}^{r}\frac{a_{i}}{N}\log\frac{a_{i}}{N}$ and $H(B)=-\sum_{j=1}^{c}\frac{b_{j}}{N}\log\frac{b_{j}}{N}$, respectively. Formally, the mutual information of two clusterings [68] can be defined as
  $$MI(A,B)=\sum_{i=1}^{r}\sum_{j=1}^{c}\frac{n_{ij}}{N}\log\frac{n_{ij}N}{a_{i}b_{j}}.$$
  The mutual information has many possible upper bounds that might be used to obtain the Normalized Mutual Information. Here, we have used max{*H*(*A*),*H*(*B*)} to normalize the MI as follows:
  (10) $$NMI=\frac{MI}{\max\left\{H,(,A,),,,H,(,B,)\right\}}.$$
2. Adjusted Rand Index (ARI): The Rand index in data clustering is a measure of the similarity between two data clusterings. The adjusted Rand Index (ARI) is defined to adjust the chance grouping of elements [69]. ARI is related to the accuracy but is applicable even when class labels are not used. It is defined as
  (11) ARI=∑ijnij2−∑iai2∑jbj2/N212∑iai2+∑jbj2−∑iai2∑jbj2/N2.

Both NMI and ARI indices change between 0 to 1, with 0 indicating that the two clusters do not agree on any pairs and 1 indicating that the clusters are exactly the same. In the following subsection, we present and compare the clustering results for the eight approaches given in Section 3.3 considering the original SCOP labels as the gold standard in four different tasks given in Section 3.4 using the above external measures. We should point out that we do not use the class labels when applying the clustering algorithms and only use the class labels for evaluation of the clustering results.

### 3.5 Results

We implemented the eight approaches introduced in Section 3.3 (NW, SW, TM-align, Yakusa, Dali, KDE, PSCDE and PSCDE(T)) on four design tasks, given in Section 3.2 as SCOP.1 to SCOP.4, and obtained the external measures between the eight different clusters and the gold standard for each task (based on the SCOP tree). The results are presented in Table 2.

Furthermore, Table 3 compares the running time and number of iterations needed to run PSCDE and PSCDE(T) in a personal Macintosh computer with 2.5 GHz Intel Core i5 and 10 GB memory. The running time of PSCDE(T) is clearly faster than PSCDE. This is due to the fact that PSCDE(T) updates the tuning parameter (*λ*) within each iteration while PSCDE runs independent Newton–Raphson iterations for each tuning parameters separately and pick the one that minimizes the AIC [36] (see e.g. Fig. 1B). It is worth to note that the web-application is approximately 3 times faster due to the higher performance of the Shiny servers.

In the remaining of this section, we emphasize some important outcomes of each task and refer the readers to the online supplementary materials for further details.

SCOP.1 (Easy Task): As it is expected, Table 2 confirms that all of the eight competing approaches do a great job in this easy task. This can be seen in the dendrogram given in the online supplementary materials (Fig. S.2) as well. The height of the vertical lines, indicates the degree of difference between branches. The longer the line, the greater the difference.
SCOP.2 (Somewhat Hard Task): The results shown in Table 2 confirm that PSCDE and PSCDE(T) methods are again competitive with the other methods on this clustering task. The PSCDE procedure mislabels only one of the structures (same as “TM-align” method), while the other six methods give perfect classification. The dendrogram associated to this task is also given in the online supplementary materials. The height of the vertical lines in Fig. S.4 suggests that our angular density-based method provides a competitive result in clear separation of the four clusters.

The difference between the results of PSCDE and PSCDE(T) in SCOP.2 makes it interesting to further compare the associated results in more details and illustrate their properties. Fig. 1 presents the results from applying the PSCDE method. The scree plot (Fig. 1A) indicates that four components can represent most of the variability among the angular densities. The AIC plot (Fig. 1B) shows a clear minimum of the AIC corresponding to the selected penalty parameter. The scatter plots (Fig. 1C and D) of coefficients in the fitted exponential family densities show that no single coefficient can separate the four classes. Neither any pairs of the coefficients can provide a good separation, but all coefficients together give some good separation. However, one of the proteins (indicated as number 2 with black color) is mislabeled and is closer to the green color cluster.

In a similar framework, Fig. 2 presents the results from applying the proposed PSCDE(T) method. Similarly, the scree plot (Fig. 2A) indicates that four components represent most of the variability among the angular densities. The penalized log-likelihood versus iterations (Fig. 2B) shows that the convergence is achieved after 37 iterations. Although, the scatter plots (Fig. 2C) and D) of coefficients in the fitted exponential family densities show that no single coefficient can separate the four classes, but the coefficients 2 and 3 can separate three classes and with coefficients 1 together give a perfect separation of four classes.

By comparing the results of Fig. 1, Fig. 2 some interesting observations were obtained. Although the information (energy) in components 1 is less (76.3%) in PSCDE(T), it does a good job in separating the classes. Furthermore, instead of running the PSCDE for 8 different tuning parameters (324 Newton–Raphson iterations that took 14.85 min to run) and then pick the optimal one that minimizes the AIC, the proposed PSCDE(T) gives even better estimates in one run (37 iterations in 1.39 min). Note that, in PSCDE(T) the tuning parameter gets updated within the Newton–Raphson iterations, which leads to obtaining the results almost 8 (number of different tuning parameters used in PSCDE) times faster than PSCDE procedure.

SCOP.3 (Hard Task): The results shown in Table 2 indicate that TM-align, Dali, PSCDE and PSCDE(T) provided the clustering results that are in total agreement with the gold standard of SCOP.3 task. While the results of Yakusa and KDE are somehow acceptable, the performances of NW and SW are poor for this clustering task. Similar to the previous two tasks, the associated dendrogram to SCOP.3 is presented in the online supplementary material (Fig. S.6). Fig. S.6 also confirms that the first two methods (NW and SW), which are motivated from pairwise sequence alignment, produced unacceptable hierarchical clustering results. While (i) Yakusa and KDE results are somehow acceptable; (ii) TM-align and Dali have no mislabeling in this case; but clearly PSCDE and PSCDE(T) have the longest vertical lines among the respective dendrograms, indicating the highest degree of difference (separation) between the branches.
SCOP.4 (Challenging Task): The results shown in Table 2 clearly indicate that five of the approaches (NW, SW, Yakusa, Dali and KDE) failed to produce acceptable results (NMI < 0.50 and ARI ≤ 0.30), while PSCDE(T) and TM-align produced the external measures (NMI and ARI) greater than 0.50. Fig. 3 provides the dendrograms for all eight approaches and confirms that TM-align and PSCDE(T) have the longest vertical lines among the respective dendrograms with acceptable degree of separation (compared with the other six approaches). It is worth to mention that TM-align incorporates an optimal alignment of the whole 3D structures, while PSCDE(T) is only a summary statistics and ignores many aspects of the protein structure.

## 4 Discussion

This paper develops an extension to a recent technique for collective estimation of multiple bivariate densities. The proposed method develops a new set of bivariate spline functions, using a tensor product approach, which can replace the bivariate B-spline functions (based on triangulation) implemented in PSCDE. The construction of the new bivariate basis function is simpler, more appealing, and can be easily extended to handle cases with more than two dimensions. While PSCDE handles the circular nature of the angular data with some artificial constraints (that extend the notion of adjacent triangles to the triangles in boundaries), the proposed method simply uses the trigonometric spline functions, that are naturally periodic. Another advantage of the new procedure is to speed up the process by updating the smoothness parameter within the Newton–Raphson iterations and avoid a grid search over the space of smoothing parameter, *λ*, which could be very expensive in time.

The estimated coefficients of the basis expansion based on PSCDE(T) provide a low-dimensional representation of the densities that can be used for visualization and clustering the densities. In general, the PSCDE(T) algorithm is faster, more appealing and interpretable in comparison to the previous approach, PSCDE.

We have applied the proposed method to four protein structural comparison tasks with different levels of difficulties. The results of these tasks show that PSCDE(T) is a new competitive method compared with existing approaches. Furthermore, the last two tasks illustrate that the PSCDE(T) can improve the efficiency of the estimated densities by borrowing strength across distributions while the non-collective estimation method of KDE does not have such ability. This improvement directly influenced the efficiency of clustering in the last two harder tasks.

We also used this method in estimating the neighbor-dependent Ramachandran distributions (the results are given in online supplementary materials), and fed those estimates into Rosetta for loop modeling application. The ultimate results showed that PSCDE(T) is competitive with other similar methods and occasionally improve the results for some hard cases. We also included, in our web application tool, the corresponding input file that contains the 800 neighbor-dependent Ramachandran densities. This can be used by the scientific community to test the quality and applicability of PSCDE(T) approach in loop modeling or any other applications that use the neighbor-dependent Ramachandran distributions (e.g. backbone-dependent rotamer library [22], [23]).

In summary, since the angular density is only a summary statistics and ignores many aspects of the protein structure, we do not expect that it always gives the best results in an arbitrary dataset. This new methodology can be used independently or as a supplement to the existing methods.

## Figures and Tables

**Table 1:** *r* × *c* contingency table *M* relating to two clustering *A* and *B*.

		B				
		*b*_1_	…	*b*_*j*_	…	*b*_*c*_
	*a*_1_	*n*_11_	…	.	…	*n*_1*c*_
	⋮	⋮		⋮		⋮
A	*a*_*i*_	.		*n*_*ij*_		.
	⋮	⋮		⋮		⋮
	*a*_*r*_	*n*_*r*1_	…	.	…	*n*_*rc*_

**Table 2:** Comparing the clustering performance of eight approaches (NW, SW, TM-align, Yakusa, Dali, KDE, PSCDE and PSCDE(T)) on four different tasks: “Easy”, “Somewhat Hard”, “Hard” and “Challenging” (SCOP.1–SCOP.4) based on Normalized Mutual Information (NMI) and Adjusted Rand Index (ARI).

Task	Measure	NW	SW	TM-align	Yakusa	Dali	KDE	PSCDE	PSCDE(T)
SCOP.1	NMI	1.00	1.00	1.00	1.00	1.00	1.00	1.00	1.00
	ARI	1.00	1.00	1.00	1.00	1.00	1.00	1.00	1.00
SCOP.2	NMI	1.00	1.00	0.93	1.00	1.00	1.00	0.93	1.00
	ARI	1.00	1.00	0.91	1.00	1.00	1.00	0.91	1.00
SCOP.3	NMI	0.47	0.32	1.00	0.86	1.00	0.87	1.00	1.00
	ARI	0.34	0.19	1.00	0.86	1.00	0.86	1.00	1.00
SCOP.4	NMI	0.48	0.48	0.71	0.29	0.44	0.39	0.56	0.64
	ARI	0.30	0.30	0.60	0.17	0.23	0.30	0.47	0.51

**Table 3:** Running time and number of iterations to achieve the final results of PSCDE and PSCDE(T) in a personal computer.

	PSCDE(T)		PSCDE	
Method	Time (min)	Iterations	Time (min)	Iterations
SCOP.1	1.24	19	11.49	176
SCOP.2	1.39	37	14.85	324
SCOP.3	1.00	18	15.19	174
SCOP.4	0.12	7	8.52	112

## References

[1] Oldfield T.J., Hubbard R.E. (1994). Analysis of *Cα* geometry in protein structures. Proteins.

[2] Laskowski R., MacArthur M.W., Moss D., Thornton J.M. (1993). Procheck: a program to check the stereochemical quality of protein structures. J Appl Crystallogr.

[3] Hooft R.W.W., Sander C., Vriend G. (1997). Objectively judging the quality of a protein structure from a Ramachandran plot. Comput Appl Biosci: CABIOS.

[4] Davis I.W., Murray L.W., Richardson J.S., Richardson D.C. (2004). Molprobity: structure validation and all-atom contact analysis for nucleic acids and their complexes. Nucleic Acids Res.

[5] Simons K.T., Bonneau R., Ruczinski I., Baker D. (1999). Ab initio protein structure prediction of CASP III targets using ROSETTA. Proteins.

[6] Hamelryck T., Kent J.T., Krogh A. (2006). Sampling realistic protein conformations using local structural bias. PLoS Comput Biol.

[7] Boomsma W., Mardia K.V., Taylor C.C., Ferkinghoff-Borg J., Krogh A., Hamelryck T. (2008). A generative, probabilistic model of local protein structure. Proc Natl Acad Sci USA.

[8] Zhao F., Peng J., Debartolo J., Freed K.F., Sosnick T.R., Xu J. (2010). A probabilistic and continuous model of protein conformational space for template-free modeling. J Comput Biol.

[9] Rohl C.A., Strauss C.E.M., Misura K.M.S., Baker D. (2004). Protein structure prediction using Rosetta. Methods Enzymol.

[10] Benkert P., Tosatto S.C.E., Schomburg D. (2008). Qmean: a comprehensive scoring function for model quality assessment. Proteins.

[11] Gao X., Xu J., Li S.C., Li M. (2009). Predicting local quality of a sequence-structure alignment. J Bioinforma Comput Biol.

[12] Archie J., Karplus K. (2009). Applying undertaker cost functions to model quality assessment. Proteins.

[13] Qiu J., Sheffler W., Baker D., Noble W.S. (2008). Ranking predicted protein structures with support vector regression. Proteins.

[14] Maadooliat M., Gao X., Huang J.Z. (2013). Assessing protein conformational sampling methods based on bivariate lag-distributions of backbone angles. Brief Bioinform.

[15] Miao X., Waddell P.J., Valafar H. (2008). Tali: local alignment of protein structures using backbone torsion angles. J Bioinforma Comput Biol.

[16] Challis C.J., Schmidler S.C. (2012). A stochastic evolutionary model for protein structure alignment and phylogeny. Mol Biol Evol.

[17] Mu Y., Nguyen P.H., Stock G. (2005). Energy landscape of a small peptide revealed by dihedral angle principal component analysis. Proteins.

[18] Altis A., Otten M., Nguyen P.H., Hegger R., Stock G. (2008). Construction of the free energy landscape of biomolecules via dihedral angle principal component analysis. J Chem Phys.

[19] Riccardi L., Nguyen P.H., Stock G. (2009). Free-energy landscape of RNA hairpins constructed via dihedral angle principal component analysis. J Phys Chem B.

[20] Altis A., Nguyen P.H., Hegger R., Stock G. (2007). Dihedral angle principal component analysis of molecular dynamics simulations. J Chem Phys.

[21] Buck M., Bouguet-Bonnet S., Pastor R.W., MacKerell A.D. (2006). Importance of the CMAP correction to the CHARMM22 protein force field: dynamics of hen lysozyme. Biom J.

[22] Bhuyan M.S.I., Gao X. (2011). A protein-dependent side-chain rotamer library. BMC Bioinforma.

[23] Shapovalov M.V., Dunbrack R.L. (2011). A smoothed backbone-dependent rotamer library for proteins derived from adaptive kernel density estimates and regressions. Structure.

[24] Ramachandran G.N., Ramakrishnan C., Sasisekharan V. (1963). Stereochemistry of polypeptide chain configurations. J Mol Biol.

[25] Mardia K.V. (1975). Statistics of directional data. J R Stat Soc Ser B Methodol.

[26] Rivest L.P. (1988). A distribution for dependent unit vectors. Comput Stand: Theory Methods.

[27] Singh H., Hnizdo V., Demchuk E. (2002). Probabilistic model for two dependent circular variables. Biometrika.

[28] Mardia K.V., Taylor C.C., Subramaniam G.K. (2007). Protein bioinformatics and mixtures of bivariate von Mises distributions for angular data. Biometrics.

[29] Pertsemlidis A., Zelinka J., Fondon J.W., Henderson R.K., Otwinowski Z. (2005). Bayesian statistical studies of the Ramachandran distribution. Stat Appl Genet Mol Biol.

[30] Dahl D.B., Bohannan Z., Mo Q., Vannucci M., Tsai J.W. (2008). Assessing side-chain perturbations of the protein backbone: a knowledge based classification of residue ramachandran space. J Mol Biol.

[31] Dunbrack R.L., Cohen F.E. (1997). Bayesian statistical analysis of protein side-chain rotamer preferences. Protein Sci.

[32] Lennox K.P., Dahl D.B., Vannucci M., Tsai J.W. (2009). Density estimation for protein conformation angles using a bivariate von Mises distribution and Bayesian nonparametrics. J Am Stat Assoc.

[33] Lennox K.P., Dahl D.B., Vannucci M., Day R., Tsai J.W. (2010). A Dirichlet process mixture of hidden Markov models for protein structure prediction. Ann Appl Stat.

[34] Ting D., Wang G., Shapovalov M., Mitra R., Jordan M.I., Dunbrack R.L. (2010). Neighbor-dependent Ramachandran probability distributions of amino acids developed from a hierarchical Dirichlet process model. PLoS Comput Biol.

[35] Joo H., Chavan A.G., Day R., Lennox K.P., Sukhanov P., Dahl D.B. (2011). Near-native protein loop sampling using nonparametric density estimation accommodating sparcity. PLoS Comput Biol.

[36] Maadooliat M., Zhou L., Najibi S.M., Gao X., Huang J.Z. (2016). Collective estimation of multiple bivariate density functions with application to angular-sampling-based protein loop modeling. J Am Stat Assoc.

[37] Schellhase C., Kauermann G. (2012). Density estimation and comparison with a penalized mixture approach. Comput Stat.

[38] Murzin A.G., Brenner S.E., Hubbard T., Chothia C. (1995). SCOP: a structural classification of proteins database for the investigation of sequences and structures. J Mol Biol.

[39] Orengo C.A., Michie A.D., Jones S., Jones D.T., Swindells M.B., Thornton J.M. (1997). CATJ — a hierarchic classification of protein domain structures. Structure.

[40] Andreeva A., Howorth D., Chandonia J.-M., Brenner S.E., Hubbard T.J.P., Chothia C. (2008). Data growth and its impact on the scop database: new developments. Nucleic Acids Res.

[41] Green P., Silverman B. (1994). Nonparametric regression and generalized linear models: a roughness penalty approach.

[42] Akaike H. (1974). A new look at the statistical model identification. IEEE Trans Autom Control.

[43] Schall R. (1991). Estimation in generalized linear models with random effects. Biometrika.

[44] Lai M., Schumaker L. (2007). Spline functions on triangulations. Number v. 13 in encyclopedia of mathematics and its applications.

[45] De Boor C. (1978). A practical guide to splines.

[46] Lyche T., Winther R. (1979). A stable recurrence relation for trigonometric-splines. J Approx Theory.

[47] Singh H., Hnizdo V., Demchuk E. (2002). Probabilistic model for two dependent circular variables. Biometrika.

[48] Mardia K.V., Taylor C.C., Subramaniam G.K. (2007). Protein bioinformatics and mixtures of bivariate von Mises distributions for angular data. Biometrics.

[49] Schumaker L.L. (1981). Spline functions: basic theory.

[50] Schumaker L.L., Traas C. (1991). Fitting scattered data on spherelike surfaces using tensor products of trigonometric and polynomial splines. Numer Math.

[51] Eilers P.H., Marx B.D. (1996). Flexible smoothing with b-splines and penalties. Stat Sci.

[52] Gough J., Karplus K., Hughey R., Chothia C. (2001). Assignment of homology to genome sequences using a library of hidden Markov models that represent all proteins of known structure. J Mol Biol.

[53] Getz G., Starovolsky A., Domany E. (2004). F2CS: FSSP to CATH and SCOP prediction server. Bioinformatics.

[54] Cui X., Gao X. (2017). K-nearest uphill clustering in the protein structure space. Neurocomputing.

[55] Rogen P., Fain B. (2003). Automatic classification of protein structure by using Gauss integrals. Proc Natl Acad Sci.

[56] Cheek S., Qi Y., Krishna S.S., Kinch L., Grishin N. (2004). SCOPmap: automated assignment of protein structures to evolutionary superfamilies. BMC Bioinf.

[57] Camoglu O., Can T., Singh A.K., Wang Y.-F. (2005). Decision tree based information integration for automated protein classification. J Bioinforma Comput Biol.

[58] Koehl P. (2001). Protein structure similarities. Curr Opin Struct Biol.

[59] Fischer D., Elofsson A., Rice D., Eisenberg D. (1996). Assessing the performance of fold recognition methods by means of a comprehensive benchmark. Pac Symp Biocomput.

[60] Huang Y., Bonett S., Kloczkowski A., Jernigan R., Wu Z. (2012). P.R.E.S.S. — an R-package for exploring residual-level protein structural statistics. J Bioinforma Comput Biol.

[61] Sam V., Tai C.-H., Garnier J., Gibrat J.-F., Lee B., Munson P. (2008). Towards an automatic classification of protein structural domains based on structural similarity. BMC Bioinformat.

[62] Needleman S.B., Wunsch C.D. (1970). A general method applicable to the search for similarities in the amino acid sequence of two proteins. J Mol Biol.

[63] Smith T.F., Waterman M.S. (1981). Identification of common molecular subsequences. J Mol Biol.

[64] Zhang Y., Skolnick J. (2005). TM-align: a protein structure alignment algorithm based on the TM-score. Nucleic Acids Res.

[65] Carpentier M., Brouillet S., Pothier J. (2005). Yakusa: a fast structural database scanning method. Proteins.

[66] Holm L., Rosenström P. (2010). Dali server: conservation mapping in 3D. Nucleic Acids Res.

[67] Core Team R. (2016). R: a language and environment for statistical computing. https://www.R-project.org/.

[68] Strehl A., Ghosh J. (March 2003). Cluster ensembles — a knowledge reuse framework for combining multiple partitions. J Mach Learn Res.

[69] Kuncheva L.I., Hadjitodorov S.T. (Oct 2004). Using diversity in cluster ensembles. 2004 IEEE International Conference on Systems, Man and Cybernetics (IEEE Cat. No.04CH37583).

