# Supplementary material for: Protein Structure Classification and Loop Modeling Using Multiple Ramachandran Distributions
Source: Comput Struct Biotechnol J. 2017 Feb 8;15:243–54. doi: 10.1016/j.csbj.2017.01.011 (PMC5331158; doi:10.1016/j.csbj.2017.01.011)
Supplement: Supplementary file 1 — Supplementary material. [file mmc1.pdf]

# Supplementary Materials for
# Protein Structure Classification and Loop Modeling Using Multiple Ramachandran Distributions

Seyed Morteza Najibi, Mehdi Maadooliat, Lan Zhou, Jianhua Z. Huang, Xin Gao

This document contains the structure of the SCOP.1 to SCOP.4 tasks in the SCOP tree that used in Section 3 of the paper. The dendrogram of each task that compare the result of eight methods (NW, SW, TM-align, Yakusa, Dali, KDE, PSCDE and PSCDE(T)) is also presented. Furthermore, another application of the proposed method in the protein loop modeling is discussed in Section S2.

# S1. More on Application: Protein Structure Classification

## S1.1 SCOP.1 (Easy Task)

Here, we considered an easy protein classification task. The goal is to classify 63 protein domains that were randomly selected from three remote *Protein* classes in SCOP. Since the class labels were not used in our exercise, this is a clustering or unsupervised learning problem. The constituents of the collection of protein domains SCOP.1 are as follows:

- 19 domains from All beta proteins/Immunoglobulin-like beta-sandwich/Immunoglobulin/V set domains (antibody variable domain-like)/Immunoglobulin light chain kappa variable domain, VL-kappa/Human (Homo sapiens), cluster 1;
- 26 domains from Alpha and beta proteins (a/b)/TIM beta, alpha-barrel/Triosephosphate isomerase (TIM)/Triosephosphate isomerase (TIM)/Triosephosphate isomerase/Chicken (Gallus gallus);
- 18 domains from Alpha and beta proteins (a+b)/Microbial ribonucleases/Microbial ribonucleases/Bacterial ribonucleases/Barnase/Bacillus amyloliquefaciens.

Here the protein domains are identified by their locations in the SCOP tree. The details of the SCOP tree involving these domains are given in Figure S.1.

Figure S.2 compares thePSCDE(T) results with other seven methods using hierarchical clustering. Clearly, there is no misclassification for this easy task by all methods.

## S1.2 SCOP.2 (Somewhat Hard Task)

In this somewhat hard task we considered 33 randomly selected domains from four *Species* under the same *Protein* subclass that belongs to the "all-alpha protein" *Class*. The constituents of the collection of domains are as follows:

- 8 domains from All-alpha proteins/Globin-like/Globin-like/Globins/Myoglobin/Sperm whale;
- 7 domains from All-alpha proteins/Globin-like/Globin-like/Globins/Myoglobin/Slug sea hare;
- 10 domains from All-alpha proteins/Globin-like/Globin-like/Globins/Myoglobin/Pig;
- 8 domains from All-alpha proteins/Globin-like/Globin-like/Globins/Myoglobin/Horse;

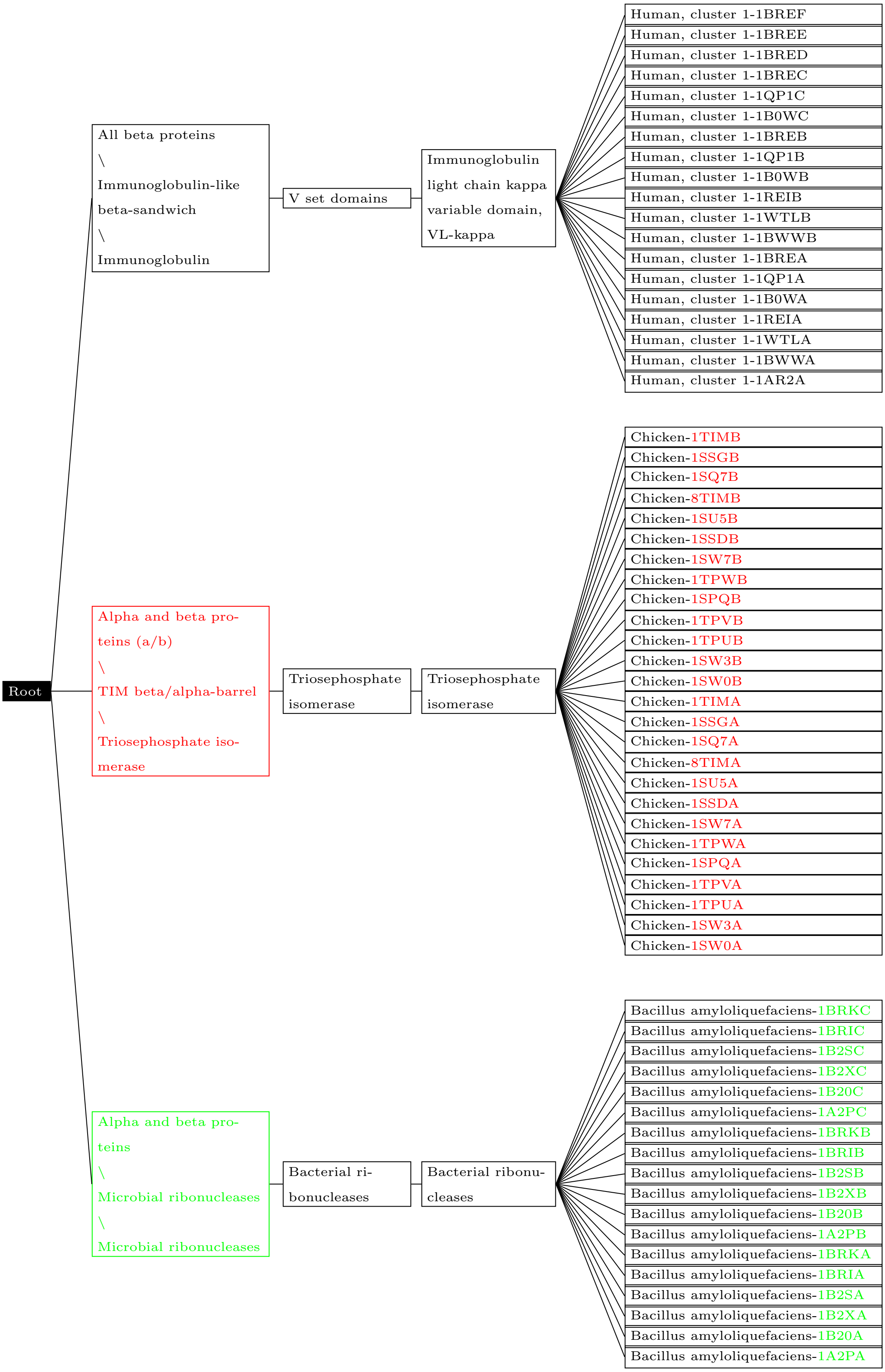


Figure S.1: SCOP.1 (Easy Task) – 63 randomly selected protein domains from SCOP tree

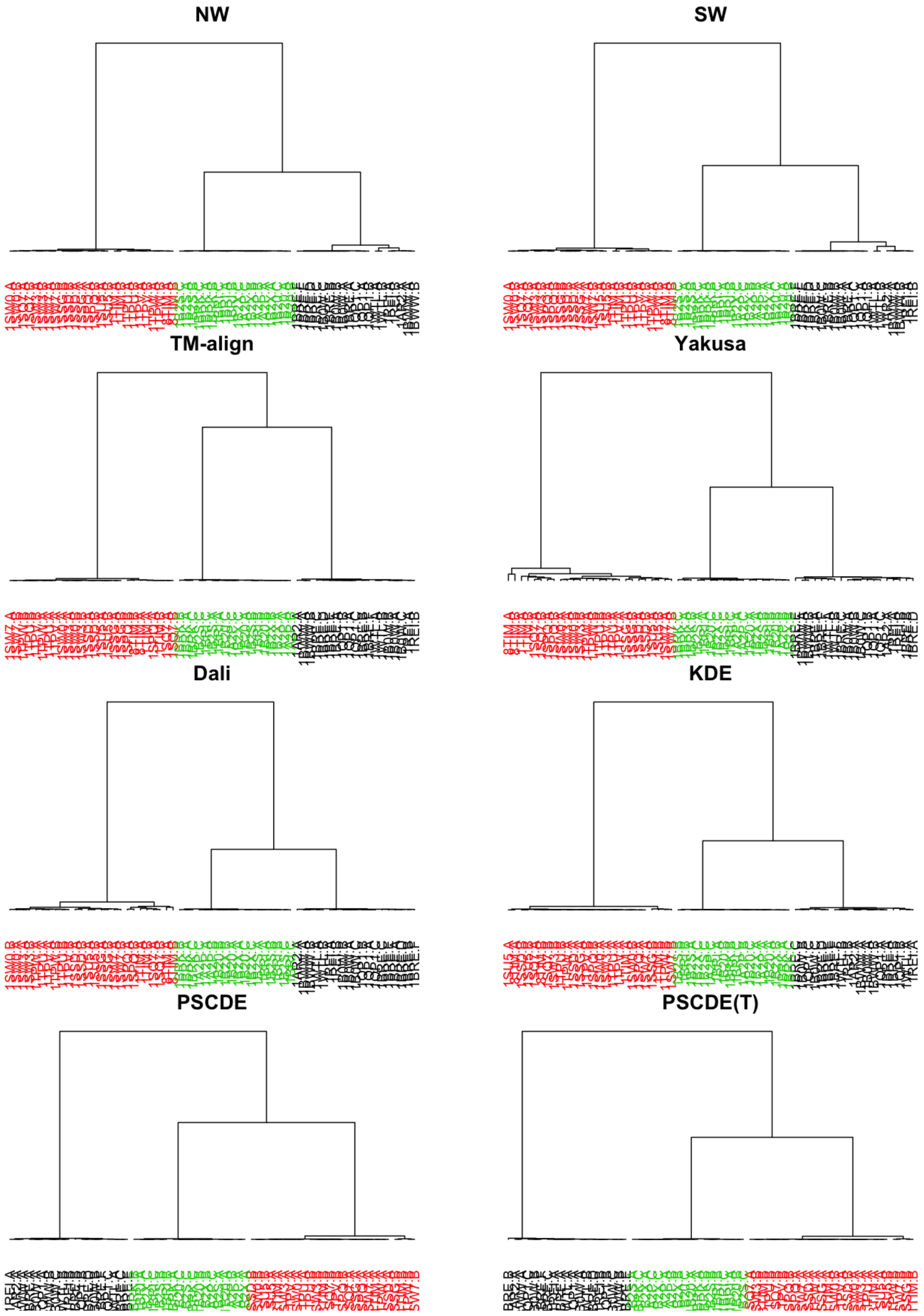


Figure S.2: SCOP.1 (Easy Task) – Dendrograms from hierarchical clustering for SCOP data.

The details of the SCOP tree involving these domains are given in Figure S.3. Figure S.4 compares the PSCDE(T) results with other seven methods using hierarchical clustering.

### S1.3 SCOP.3 (Hard Task)

We considered a protein classification task for which 40 protein chains were randomly selected from three different *Fold/Superfamily* levels, where all chains belong to the "Alpha and beta proteins (a+b)" *Class*. The constituents of the collection of domains are as follows:

- 12 domains from Microbial ribonucleases/Microbial ribonucleases/Bacterial ribonucleases;
- 14 domains from Gelsolin-like/Actin depolymerizing proteins/Gelsolin-like;
- 14 domains from Prion-like/Prion-like/Prion-like/Prion protein domain;

The details of the SCOP tree involving these domains are given in Figure S.5. Figure S.6 compares the PSCDE(T) results with other seven methods using hierarchical clustering.

### S1.4 SCOP.4 (Challenging Task)

We have selected 26 protein chains from Fischer et al. [6] benchmark in the "All beta proteins" *Class* within three different *Folds*. The constituents of the collection of domains are as follows:

- 7 domains from Immunoglobulin-like beta-sandwich/Immunoglobulin;
- 8 domains from Trypsin-like serine proteases/Trypsin-like serine proteases;
- 11 domains from Cupredoxin-like/Cupredoxins;

The details of the SCOP tree involving these domains are given in Figure S.7.

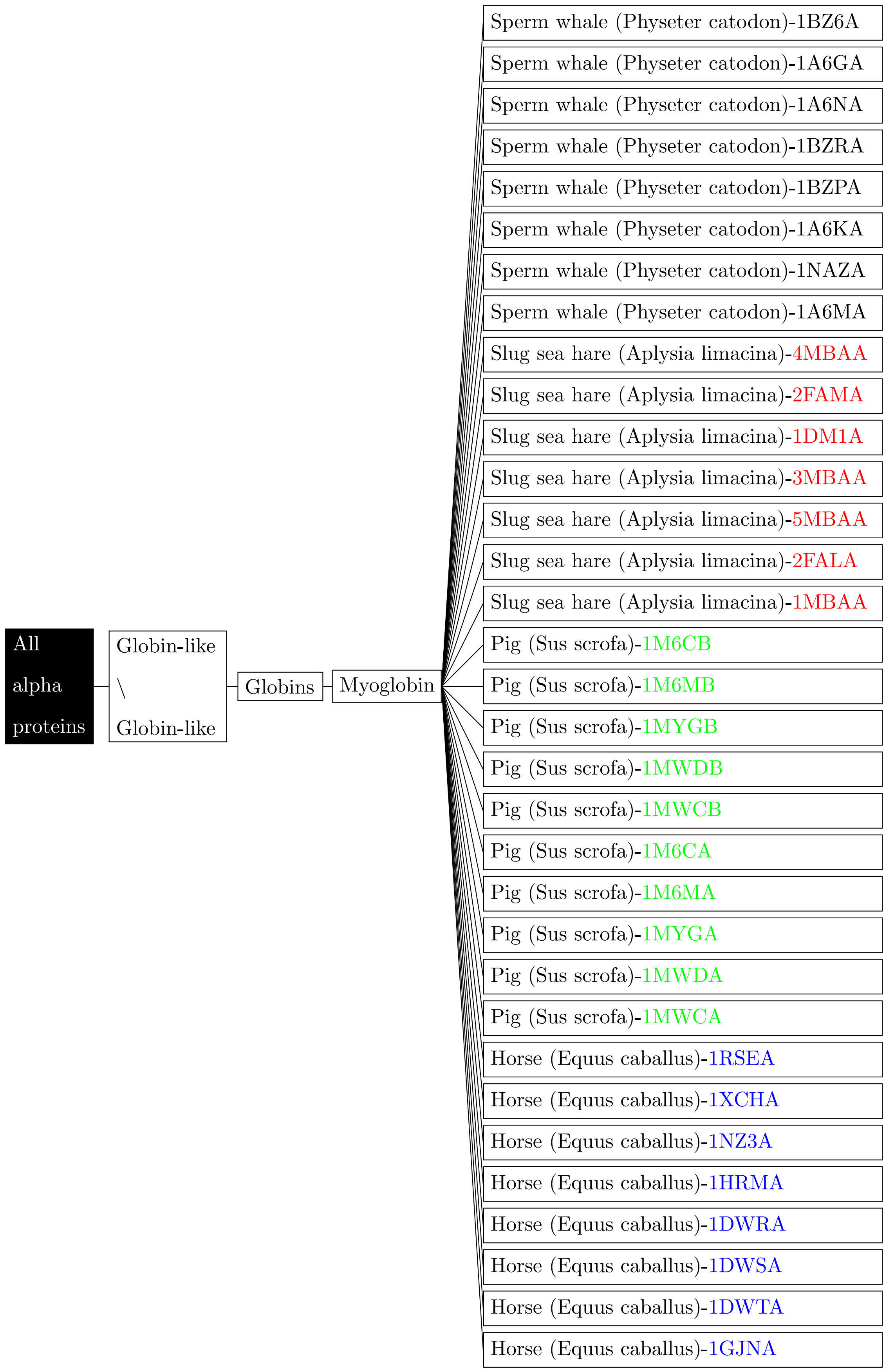


Figure S.3: SCOP.2 (Somewhat Hard Task) – 33 randomly selected protein domains from SCOP tree

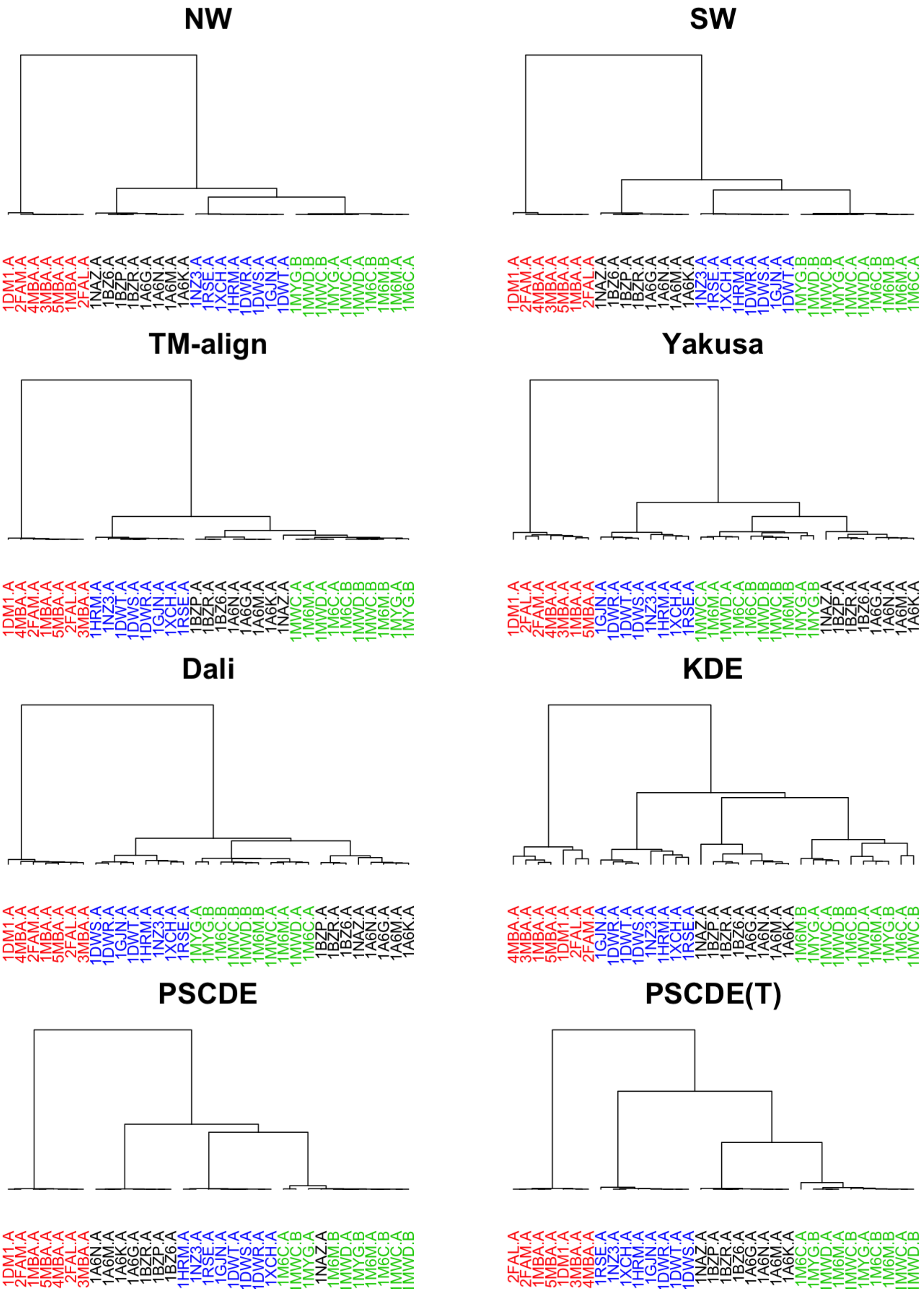


Figure S.4: SCOP.2 (Somewhat Hard Task) – Dendrograms from hierarchical clustering for SCOP data.

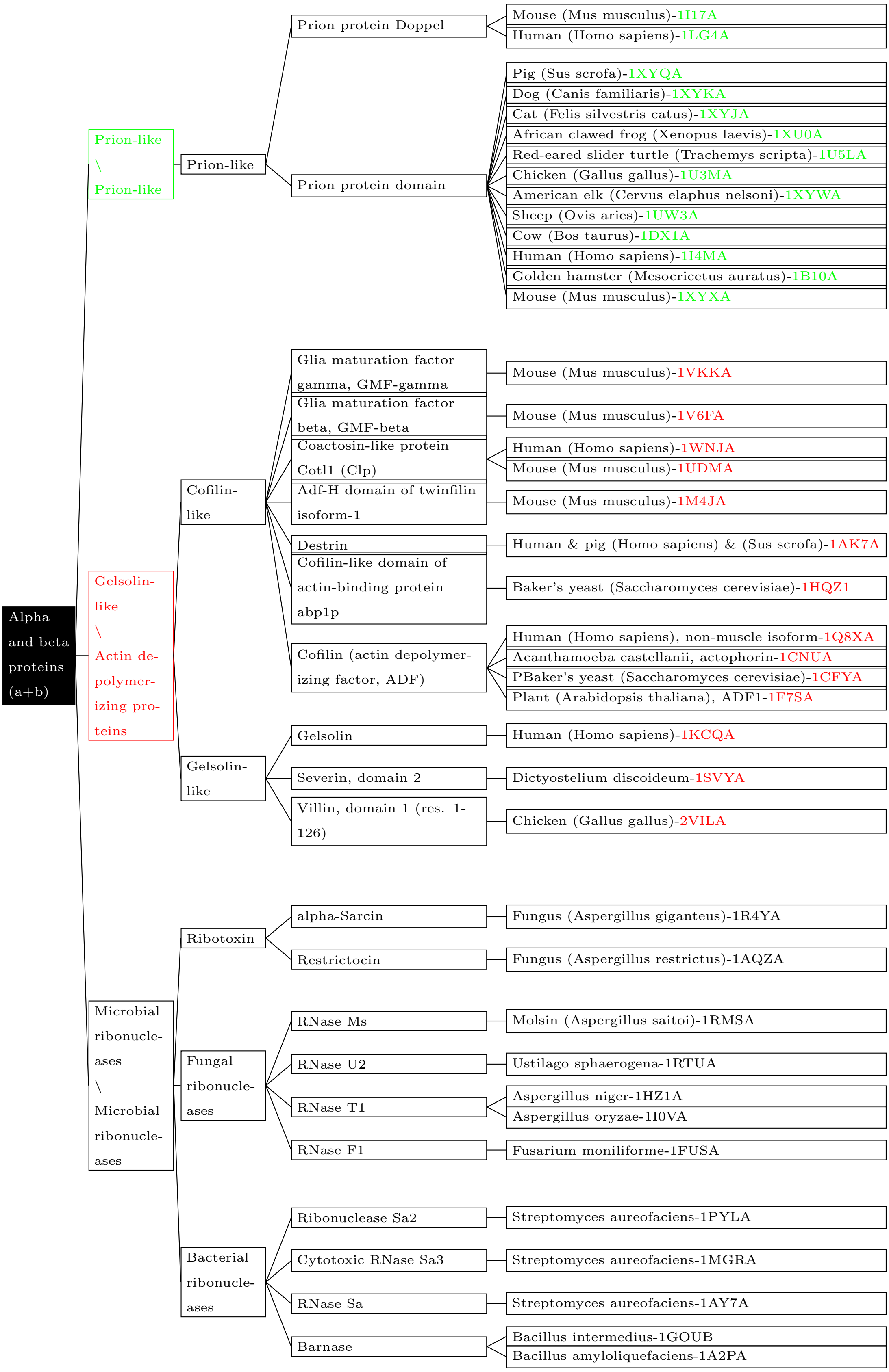


Figure S.5: SCOP.3 (Hard Task) – 40 randomly selected protein domains from SCOP tree

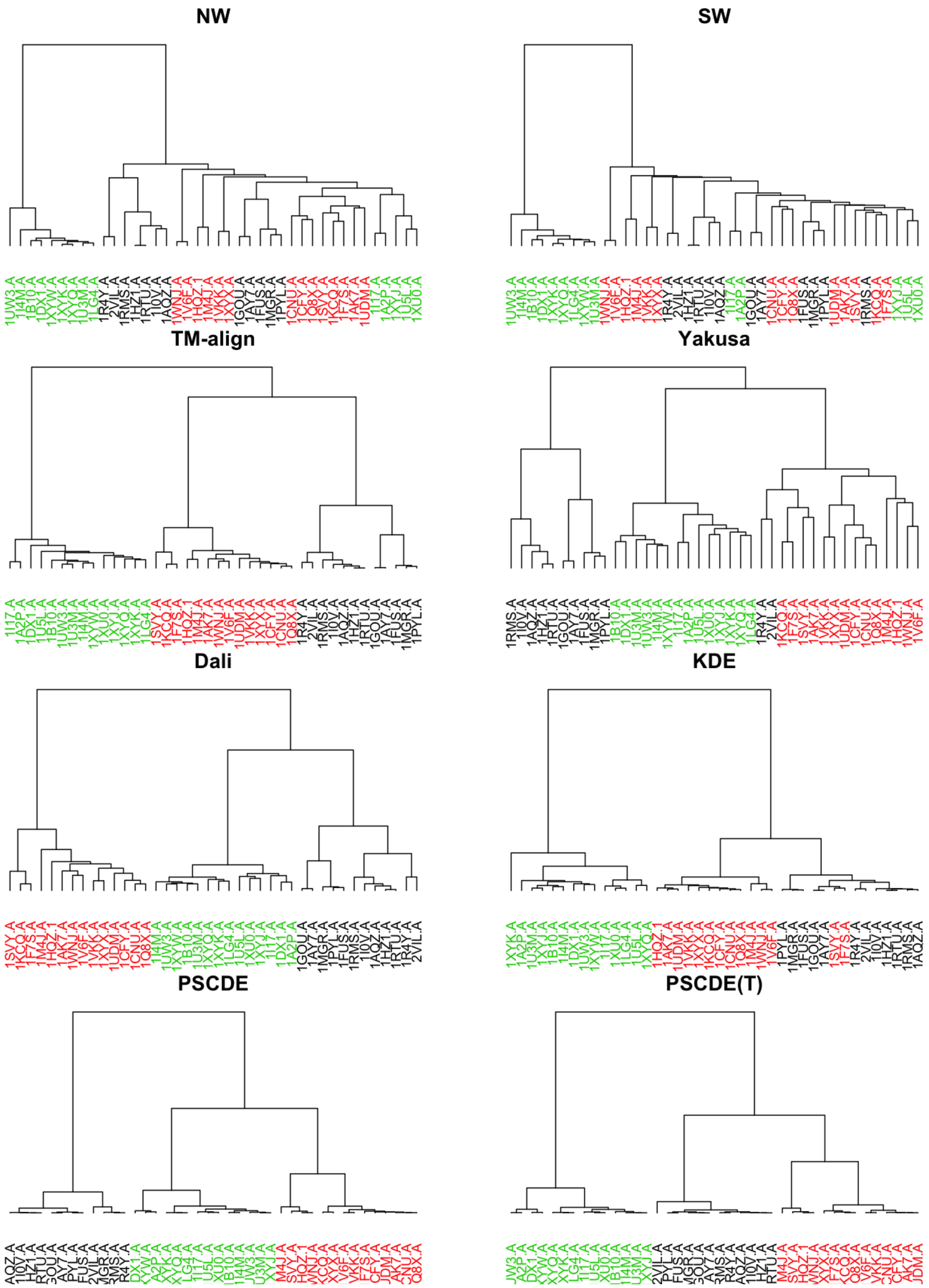


Figure S.6: SCOP.2 (Hard Task) – Dendrograms from hierarchical clustering for SCOP data.

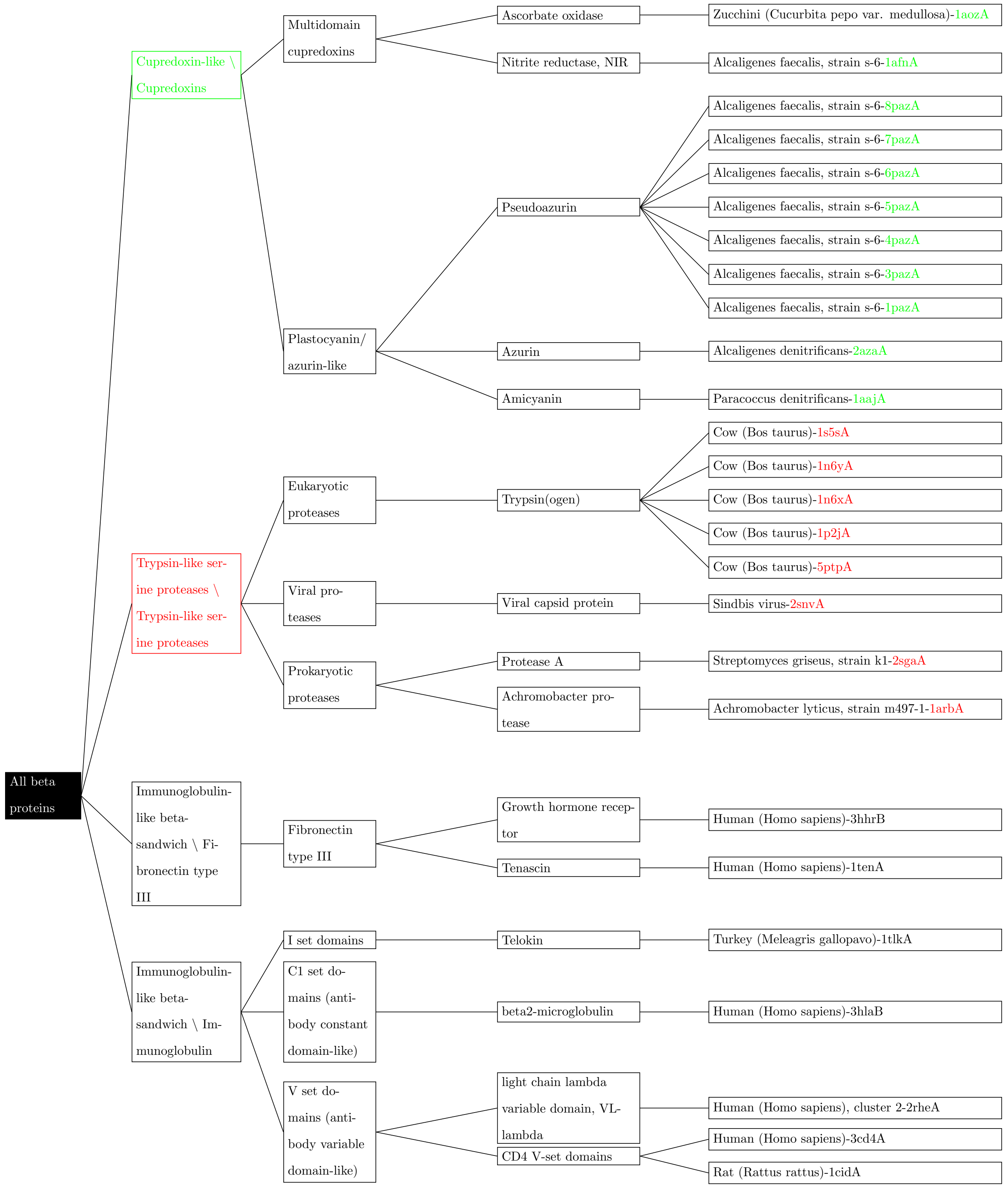


Figure S.7: SCOP.4 (Challenging Task) – 26 randomly selected protein domains from SCOP tree

# S2. Protein Loop Modeling Application

## S2.1 Loop Modeling in Literature

Template-based modeling is the most accurate technique in predicting rigid protein structures [23]. The most flexible parts of structures that cannot be easily modeled even with close structural homologs are loops [7]. The loops are the irregular part of proteins that usually join the elements of regular secondary structures. There are two strategies available in the literature to predict loop structures, *de novo* and knowledge-based loop modeling methods. The programs that use *de novo* approach samples sterically feasible loop conformations and select the best one based on their energy conformations score [5, 14, 24]. Different types of methods such as molecular dynamics simulations [8], simulated annealing [7], angular sampling of Ramachandran plots [3, 10, 12, 18, 22, 24, 27, 28, 31], and random tweaks [25, 30] are using the *de novo* approach to predict the loop regions. In contrast, the knowledge-based methods use known structures or fragments to sample the loop conformations [17, 26, 29]; but they are limited to sampling within the domain of knowledge [12].

While different approaches use different techniques, the main idea is to efficiently sample the continuous conformational space for loops and then score the samples using various energy functions [2]. An accurate energy function is often specified as a weighted linear combination of a number of statistical and empirical terms, such as terms that encode bond lengths, bond angles, torsion angles, van-der-Waals interactions, and electrostatic interactions. For instance, the Rosetta program has a scoring function based on Logarithm of Ramachandran probability densities. Most of the angular-sampling-based methods use Ramachandran distributions of 20 popular amino acids for sampling the dihedral angles. However, Ramachandran distributions are known to be affected by the secondary structure [9, 11] and the amino acid type [1] of the residue from which $\phi$ and $\psi$ angles are calculated, as well as the neighboring amino acids [13, 19, 21, 28].

## S2.2 Rosetta Loop Modeling

Among existing protein structure prediction software, Rosetta is one of the most accurate and commonly used. It provides a flexible library of functionality to accomplish a diverse set of biomolecular modeling tasks. The kinematic inversion closure (KIC) protocol in Rosetta was

developed by Mandell et al. [22] to reconstruct high-resolution loop structures. In KIC, the torsion angles, $(\phi, \psi)$, are sampled from an estimated Ramachandran distribution of the associated amino acids to explore the conformational space effectively. The sampling step is followed by a Monte-Carlo minimization step that involves an empirical energy function. Mandell et al. [22] demonstrated that Rosetta with this KIC procedure could accurately predict native-like structures of protein loop regions. Following the promising results of the KIC in obtaining accurate predictions for the local protein structures, Stein and Kortemme [27] developed a new protocol, called "next-generation KIC" (NGK), to improve Rosetta's KIC protocol further. Their procedure consists of a combination of 4 different strategies to improve the percentage of reconstructed loops that are within $1\mathring{A}$ to the native structure.

Here, we focus on the intensification strategy whose aim is to intensify sampling of $(\phi, \psi)$ from certain regions of neighbor-dependent Ramachandran distributions (referred to as Rama2b sampling). The neighbor-dependent Ramachandran distributions used in NGK were provided by fitting a hierarchical Dirichlet process (HDP) model [28]. From here on we refer to the Stein and Kortemme procedure as NGK.HDP. To assess the direct effect of Rama2b sampling on KIC, we also consider the Rama2b strategies in the KIC protocol which from here on we refer to as KIC.HPD. Following Maadooliat et al. [20], we replace the neighbor-dependent Ramachandran distributions in Rosetta obtained by HDP by the ones we obtain from the PSCDE and PSCDE(T) and refer to the new methods as KIC.PSCDE, KIC.PSCDE(T), NGK.PSCDE and NGK.PSCDE(T). To facilitate a fair comparison, all other components of NGK remain the same. We evaluate the performance of different density estimation methods using the task of protein loop structure prediction. In our comparison, we also included Rosetta's KIC protocol as a benchmark.

### S2.3 Methodology

We used the same dataset provided in Ting et al. [28] to obtain the neighbor-dependent Ramachandran distributions when applying the proposed PSCDE(T) method. The data set consisted of $3,038$ proteins with available electron densities from the Uppsala Electron Density Server [15]. As in Ting et al. [28], we considered a set of $62,345$ residues after removing those with electron density in the bottom $20^{th}$ percentile and restricting the set to loop residues with no missing backbone atoms and at least three residues away from $\alpha$-helices or $\beta$-sheets. For each amino acid type, we applied PSCDE(T) (with $K = 4$) to collectively estimate the $m = 20$

left-neighbor-dependent Ramachandran distributions; we also applied PSCDE(T) to collectively estimate the $m = 20$ right-neighbor-dependent Ramachandran distributions. Since there are 20 possible amino acid types, we obtained 800 ($= 20 \times (20 + 20)$) neighbor-dependent estimated density functions. The number of data points available for each of these 800 density functions, ranges from 6 to 620, with the median 131 and quantiles 68.75 and 213.20. In addition, we partition the range of $\phi$ and $\psi$ (from $-\pi$ to $\pi$) to 90 equidistant grid points and evaluate the bivariate log-densities in those $90 \times 90$ grid points. Furthermore, we use 12 knots ($\kappa$) to construct the trigonometric spline bases in each direction with order $\nu = 3$.

We assessed the seven methods ( KIC, KIC.HPD, KIC.PSCDE, KIC.PSCDE(T), NGK.HDP, NGK.PSCDE, NGK.PSCDE(T) ) by reconstructing the structures of short loops (12-residue segments) from an established benchmark dataset with 20 proteins. This benchmark was compiled by Zhu et al. [32] to allow direct comparison to studies by Jacobson et al. [10], Zhu et al. [32], and Sellers et al. [24]. It was selected from high quality structures (resolution $\leq 2.0\mathring{A}$, R $< 0.25$) for loops with diverse sequences ($< 40\%$ sequence identity), low temperature factors ($< 35$), lack of contacts to heteroatom groups ($> 4.0\mathring{A}$ for neutral ligands, $> 6.5\mathring{A}$ for metal ions), lack of secondary structure within the loop, lack of more than 4 loop residues adjacent to either loop endpoint, and pH 6.5–7.5; see Mandell et al. [22] for more details.

Following Stein and Kortemme [27], we used two metrics to evaluate the performance of each method: The first metric is the percentage of reconstructed loops that are within $1\mathring{A}$ to the native structure (i.e., sub-angstrom cases), denoted as $\%sA$. The second metric is the lowest root mean square deviation ($RMSD$) of the backbone atoms between the 10 lowest energy reconstructed loops and the native structure, denoted as $RMSD^*$. The first metric measures the ability to sample high-quality loops, whereas the second metric measures the ability to select the best loops within the simulated ones based on the associated energy functions.

The following pre-processing step was needed before we proceed with the reconstruction procedure. Each structure was prepared by first removing all native side-chain information (including side-chain bond lengths, bond angles, and chi angles) and then replacing them with rotameric conformations from the Dunbrack backbone-dependent rotamer library [4] and ideal bond lengths and angles. These rotamers were then simultaneously optimized by Metropolis Monte Carlo (MC) simulated annealing (repacking) using Rosetta, as described in Kuhlman et al. [16]. After this preparation procedure, the loop segment of each protein is deleted from the pro-

tein structure and then "reconstructed" using KIC, KIC.HPD, KIC.PSCDE, KIC.PSCDE(T), NGK.HDP, NGK.PSCDE, NGK.PSCDE(T) methods, respectively. Although KIC and NGK.HDP have demonstrated considerable success in sampling and correctly identifying near-native conformations on this benchmark [20, 27]; for some of the proteins, sub-Angstrom conformations were either not sampled or not identified correctly by the energy function.

## S2.4 Results

For each of the 20 benchmark proteins, we reconstructed 500 loop structures for the associated assigned loop (12-residue) using different methods. The results are summarized in Table S.1. For "1BN8," none of the methods were able to generate any structure predictions (probably due to some internal issues of Rosetta), so both $\%sA$ and $RMSD^*$ are unavailable for this protein. For two out of the rest of 19 proteins ("1CNV" and "1CS6"), we did not obtain any sub-angstrom structure using any of the seven methods, as indicated by $\%sA$ being 0.0.

Table S.1 shows that KIC and KIC.HPD, each obtains the highest $\%sA$ for one protein ("1MS9" and "1M3S", respectively). While KIC.PSCDE does not achieve any of the best scores for $\%sA$, KIC.PSCDE(T) gets the highest $\%sA$ for 4 of the proteins ("1I7P," "1ARB," "1DQZ" and "1MY7"). Therefore, KIC.PSCDE(T) is the best among four variates of KIC methods with respect to the percentage of sub-angstrom structures criterion on this benchmark. Furthermore, the NGK.HPD, NGK.PSCDE and NGK.PSCDE(T) obtain the highest $\%sA$ for 4, 4 and 3 proteins, respectively. Although the three NGK methods are overall comparable, the NGK.PSCDE and NGK.PSCDE(T) seem to be superior for the harder cases (best $\%sA < 30\%$) with respect to the percentage of sub-angstrom structures criterion on this benchmark.

Another observation from Table S.1 is that all of the methods except KIC.PSCDE(T) obtain the highest $\%sA$ and smallest $RMSD^*$, simultaneously, for at most one protein; whereas for the KIC.PSCDE(T) for 3 proteins ("1I7P," "1DQZ" and "1MY7") we obtain the highest $\%sA$ and the minimum $RMSD^*$. This is a significant result, since a method that increases the chance of $\%sA$ and reduces the minimum $RMSD^*$, at the same time, is practically desirable.

Figure S.8, compares the RMSD and Energy of associated 500 reconstructed loops for the protein "1MY7" among 7 different techniques (KIC, KIC.HPD, KIC.PSCDE, KIC.PSCDE(T), NGK.HDP, NGK.PSCDE, NGK.PSCDE(T)). Native-like percentage values provide the percentage of the sub-angstrom results for each method (out of associated 500 reconstructed loops). By using this percentage criterion, KIC.HDP and KIC.PSCDE did not obtain any better results

than KIC, while our proposed KIC.PSCDE(T) method improved the sub-angstrom percentage by 7% comparing to KIC in this protein (see Figure S.8(a-d)). Furthermore, the three NGK methods did not improve the KIC results (see Figure S.8(e-g)).

| | KIC | | KIC.HDP | | KIC.PSCDE | | KIC.PSCDE(T) | | NGK.HDP | | NGK.PSCDE | | NGK.PSCDE(T) | |
|---|---|---|---|---|---|---|---|---|---|---|---|---|---|---|
| | %sA | RMSD* | %sA | RMSD* | %sA | RMSD* | %sA | RMSD* | %sA | RMSD* | %sA | RMSD* | %sA | RMSD* |
| 1BN8 | - | - | - | - | - | - | - | - | - | - | - | - | - | - |
| 1CNV | 0.00 | 1.82 | 0.00 | 1.48 | 0.00 | 1.89 | 0.00 | 1.45 | 0.00 | 1.37 | 0.00 | **1.20** | 0.00 | 1.40 |
| 1F46 | 0.00 | 2.43 | 0.00 | 2.43 | 0.00 | 2.06 | 0.00 | 2.45 | 7.00 | 2.14 | **9.60** | 1.05 | 7.40 | **1.04** |
| 1CS6 | 0.00 | 3.20 | 0.00 | 2.50 | 0.00 | 3.58 | 0.00 | 1.77 | 0.00 | 2.62 | 0.00 | **1.14** | 0.00 | 2.66 |
| 1A8D | 0.40 | 0.45 | 0.20 | 0.51 | 0.20 | 0.48 | 0.20 | 0.45 | 1.00 | 0.44 | 1.40 | 0.37 | **1.80** | **0.36** |
| 1OYC | 0.40 | 0.70 | 3.40 | 0.37 | 0.80 | 0.36 | 3.40 | 0.35 | 19.20 | 0.30 | **23.20** | **0.28** | 21.6 | 0.31 |
| 1QLW | 1.40 | 0.50 | 3.00 | 0.64 | 5.20 | 0.57 | 3.20 | 0.71 | 10.00 | **0.35** | **13.80** | 0.42 | 10.40 | 0.55 |
| 1BHE | 3.20 | 0.41 | 3.20 | 0.34 | 3.20 | 0.38 | 1.60 | 0.41 | 5.80 | **0.25** | 6.00 | 0.26 | **6.60** | 0.29 |
| 1T1D | 4.20 | 0.82 | 4.40 | 0.82 | 4.00 | 0.82 | 7.20 | 0.83 | 12.40 | **0.49** | **15.20** | 0.54 | 11.80 | 0.60 |
| 1I7P | 11.60 | 0.38 | 11.20 | 0.38 | 12.20 | 0.39 | **14.20** | **0.37** | 7.40 | **0.37** | 7.00 | 0.38 | 8.20 | **0.37** |
| 1ARB | 11.60 | 0.66 | 5.80 | 0.54 | 2.60 | 0.62 | **16.4** | 0.56 | 24.40 | 0.51 | 14.40 | **0.42** | 21.0 | 0.49 |
| 1OTH | 20.00 | 0.57 | 5.20 | 0.46 | 7.60 | 0.52 | 7.20 | 0.65 | 18.20 | **0.30** | 27.20 | 0.33 | **29.00** | 0.34 |
| 1M3S | 20.40 | 2.40 | **48.20** | **1.16** | 19.20 | 2.43 | 46.40 | 2.95 | 34.80 | 2.16 | 21.80 | 2.93 | 31.40 | 2.80 |
| 1C5E | 27.60 | 0.41 | 25.60 | 0.45 | 14.20 | 0.44 | 21.01 | 0.45 | **41.40** | 0.37 | 35.02 | 0.37 | 38.80 | **0.35** |
| 1DQZ | 29.20 | 0.33 | 43.20 | 0.33 | 32.80 | 0.34 | **40.60** | **0.31** | 33.40 | 0.37 | 22.80 | 0.33 | 24.40 | 0.34 |
| 1CB0 | 31.60 | 0.49 | 48.05 | 0.51 | 18.60 | 0.40 | 34.20 | 0.48 | **50.20** | 0.41 | 32.61 | **0.35** | 45.61 | 0.41 |
| 1EXM | 43.80 | 0.72 | 38.20 | 0.71 | 28.41 | 0.49 | 41.62 | **0.41** | **60.60** | 0.52 | 57.21 | 0.44 | 58.82 | 0.51 |
| 1MS9 | **67.60** | 0.30 | 47.36 | 0.31 | 66.00 | 0.31 | 54.40 | 0.35 | 40.40 | 0.34 | 49.40 | **0.29** | 41.22 | 0.36 |
| 1MY7 | 70.80 | 0.45 | 55.60 | 0.48 | 30.40 | 0.46 | **77.80** | **0.40** | 47.60 | 0.49 | 45.0 | 0.45 | 63.0 | 0.47 |
| 2PIA | 94.07 | 0.87 | 96.40 | 0.84 | 96.03 | 0.86 | 95.20 | 0.86 | **97.04** | **0.63** | 96.80 | 0.78 | 96.20 | 0.80 |

Table S.1: Comparing the performance of KIC, KIC.HPD, KIC.PSCDE, KIC.PSCDE(T), NGK.HDP, NGK.PSCDE and NGK.PSCDE(T) for reconstructing short loops with the length of 12-residue for 20 benchmark proteins based on 500 simulation runs. For each of the benchmark proteins, the method produces the highest percentage of sub-angstrom structures ($\%sA$) is denoted as bold. Similarly, the method that produces the smallest $RMSD^*$ on the energy score is indicated in bold.

Finally, we investigate the distribution of RMSD among the KIC protocols ( KIC, KIC.HPD, KIC.PSCDE, KIC.PSCDE(T) ). In Figure S.9, the side-by-side boxplots of RMSD for all simulated loops in three proteins ("1MY7","1ARB" and "1I7P") and the whole benchmark set are plotted. The result shows that, e.g., in protein "1MY7" the distributions of RMSD in KIC.PSCDE(T) is better than the other methods, while in proteins "1I7P", the distributions are almost the same. In summary, Figure S.9 and some similar investigations in the benchmark set show that the proposed procedure, KIC.PSCDE(T) and NGK.PSCDE(T) methods produce

competitive results in comparing with the existing methods, and there are some cases that the new procedures outperform.

## References


[1] Berkholz, D. S., Shapovalov, M. V., Dunbrack, Jr, R. L., and Karplus, P. A. (2009), "Conformation dependence of backbone geometry in proteins," *Structure*, 17, 1316–1325.

[2] Bruccoleri, R. E. and Karplus, M. (1987), "Prediction of the folding of short polypeptide segments by uniform conformational sampling," *Biopolymers*, 26, 137–168.

[3] Deane, C. M. and Blundell, T. L. (2000), "A novel exhaustive search algorithm for predicting the conformation of polypeptide segments in proteins," *Proteins: Structure, Function, and Bioinformatics*, 40, 135–144.

[4] Dunbrack, R. L. and Cohen, F. E. (1997), "Bayesian statistical analysis of protein side-chain rotamer preferences," *Protein Science*, 6, 1661–1681.

[5] Felts, A. K., Gallicchio, E., Chekmarev, D., Paris, K. A., Friesner, R. A., and Levy, R. M. (2008), "Prediction of protein loop conformations using the AGBNP implicit solvent model and torsion angle sampling," *Journal of chemical theory and computation*, 4, 855–868.

[6] Fischer, D., Elofsson, A., Rice, D., and Eisenberg, D. (1996), "Assessing The Performance Of Fold Recognition Methods By Means Of A Comprehensive Benchmark," *Pacific Symposium on Biocomputing*, 300–318.

[7] Fiser, A., Do, R. K. G., and Sali, A. (2000), "Modeling of loops in protein structures," *Protein Science*, 9, 1753–1773.

[8] Hornak, V. and Simmerling, C. (2003), "Generation of accurate protein loop conformations through low-barrier molecular dynamics," *Proteins: Structure, Function, and Bioinformatics*, 51, 577–590.

[9] Hovmöller, S., Zhou, T., and Ohlson, T. (2002), "Conformations of amino acids in proteins," *Acta Crystallogr D Biol Crystallogr*, 58, 768–776.

[10] Jacobson, M. P., Pincus, D. L., Rapp, C. S., Day, T. J., Honig, B., Shaw, D. E., and Friesner, R. A. (2004), "A hierarchical approach to all-atom protein loop prediction," *Proteins: Structure, Function, and Bioinformatics*, 55, 351–367.

[11] Jha, A., Colubri, A., Zaman, M., Koide, S., Sosnick, T., and Freed, K. (2005), "Helix, sheet, and polyproline II frequencies and strong nearest neighbor effects in a restricted coil library," *Biochemistry*, 44, 9691–9702.

[12] Joo, H., Chavan, A. G., Day, R., Lennox, K. P., Sukhanov, P., Dahl, D. B., Vannucci, M., and Tsai, J. (2011), "Near-native protein loop sampling using nonparametric density estimation accommodating sparcity," *PLoS computational biology*, 7, e1002234.

[13] Keskin, O., Yuret, D., Gursoy, A., Turkay, M., and Erman, B. (2004), "Relationships between amino acid sequence and backbone torsion angle preferences," *Proteins*, 55, 992–998.

[14] Kim, D. E., Blum, B., Bradley, P., and Baker, D. (2009), "Sampling bottlenecks in de novo protein structure prediction," *Journal of molecular biology*, 393, 249–260.

[15] Kleywegt, G. J., Harris, M. R., Zou, J.-y., Taylor, T. C., Wälby, A., and Jones, T. A. (2004), "The Uppsala Electron-Density Server," *Acta Crystallographica Section D*, 60, 2240–2249.

[16] Kuhlman, B., Dantas, G., Ireton, G. C., Varani, G., Stoddard, B. L., and Baker, D. (2003), "Design of a novel globular protein fold with atomic-level accuracy," *Science*, 302, 1364–1368.

[17] Lee, J., Lee, D., Park, H., Coutsias, E. A., and Seok, C. (2010), "Protein loop modeling by using fragment assembly and analytical loop closure," *Proteins: Structure, Function, and Bioinformatics*, 78, 3428–3436.

[18] Lennox, K. P., Dahl, D. B., Vannucci, M., Day, R., and Tsai, J. W. (2010), "A Dirichlet Process Mixture of Hidden Markov Models for Protein Structure Prediction," *Ann Appl Stat*, 4, 916–942.

[19] Lennox, K. P., Dahl, D. B., Vannucci, M., and Tsai, J. W. (2009), "Density Estimation for Protein Conformation Angles Using a Bivariate von Mises Distribution and Bayesian Nonparametrics," *Journal of the American Statistical Association*, 104, 586–596.

[20] Maadooliat, M., Zhou, L., Najibi, S. M., Gao, X., and Huang, J. Z. (2016), "Collective Estimation of Multiple Bivariate Density Functions With Application to Angular-Sampling-Based Protein Loop Modeling," *Journal of the American Statistical Association*, 111, 43–56.

[21] MacArthur, M. W. and Thornton, J. M. (1991), "Influence of proline residues on protein conformation," *Journal of molecular biology*, 218, 397–412.

[22] Mandell, D. J., Coutsias, E. A., and Kortemme, T. (2009), "Sub-angstrom accuracy in protein loop reconstruction by robotics-inspired conformational sampling," *Nature methods*, 6, 551–552.

[23] Qu, X., Swanson, R., Day, R., and Tsai, J. (2009), "A guide to template based structure prediction," *Current Protein and Peptide Science*, 10, 270–285.

[24] Sellers, B. D., Zhu, K., Zhao, S., Friesner, R. A., and Jacobson, M. P. (2008), "Toward better refinement of comparative models: predicting loops in inexact environments," *Proteins: Structure, Function, and Bioinformatics*, 72, 959–971.

[25] Shenkin, P. S., Yarmush, D. L., Fine, R. M., Wang, H., and Levinthal, C. (1987), "Predicting antibody hypervariable loop conformation. I. Ensembles of random conformations for ringlike structures," *Biopolymers*, 26, 2053–2085.

[26] Soto, C. S., Fasnacht, M., Zhu, J., Forrest, L., and Honig, B. (2008), "Loop modeling: sampling, filtering, and scoring," *Proteins: Structure, Function, and Bioinformatics*, 70, 834–843.

[27] Stein, A. and Kortemme, T. (2013), "Improvements to robotics-inspired conformational sampling in Rosetta," *PloS one*, 8, e63090.

[28] Ting, D., Wang, G., Shapovalov, M., Mitra, R., Jordan, M. I., and Dunbrack, Jr, R. L. (2010), "Neighbor-dependent Ramachandran probability distributions of amino acids developed from a hierarchical Dirichlet process model," *PLOS Computational Biology*, 6, e1000763.

[29] Wojcik, J., Mornon, J.-P., and Chomilier, J. (1999), "New efficient statistical sequence-

dependent structure prediction of short to medium-sized protein loops based on an exhaustive loop classification," *Journal of molecular biology*, 289, 1469–1490.

[30] Xiang, Z., Soto, C. S., and Honig, B. (2002), "Evaluating conformational free energies: the colony energy and its application to the problem of loop prediction," *Proceedings of the National Academy of Sciences*, 99, 7432–7437.

[31] Zhao, F., Peng, J., Debartolo, J., Freed, K. F., Sosnick, T. R., and Xu, J. (2010), "A probabilistic and continuous model of protein conformational space for template-free modeling." *J Comput Biol*, 17, 783–798.

[32] Zhu, K., Pincus, D. L., Zhao, S., and Friesner, R. A. (2006), "Long loop prediction using the protein local optimization program," *Proteins: Structure, Function, and Bioinformatics*, 65, 438–452.

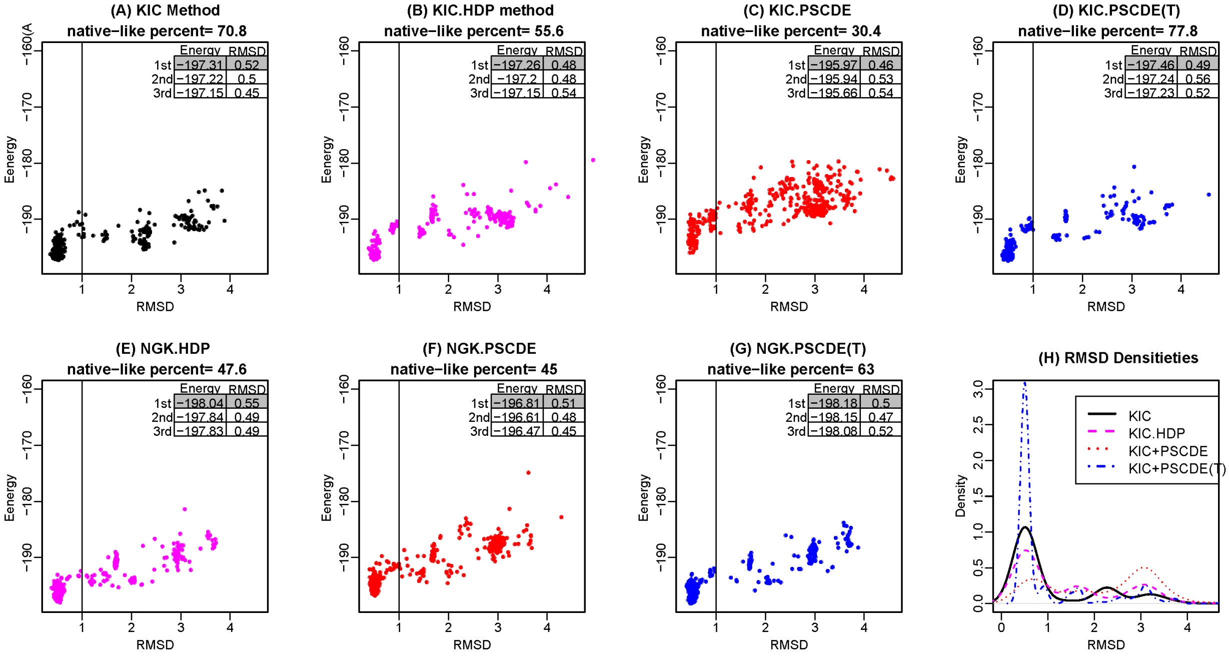


Figure S.8: Plots of RMSD versus Rosetta Energy Unit(REU) for protein “1MY7” based on KIC and NGK with different methods of density estimation of neighbor-dependent of Ramachandran distributions. (a) KIC method that simulated 70.8% loops in sub-angstrom level; (b) KIC.HDP method [28] which despite adding neighboring information, fraction of native-like is less than standard KIC (55.6%); (c) KIC.PSCDE [20] that the percent of native-like is 30.4%; (d) KIC.PSCDE(T) which the native-like percentage is the highest (77.8%); (e-g) Result of NGK.HDP, NGK.PSCDE and NGK.PSCDE(T) methods; (h) Kernel density estimates of RMSD for KIC, KIC.HDP, KIC.PSCDE and KIC.PSCDE(T). The RMSD density associated to KIC.PSCDE(T) is significantly different from the other methods, and the peak of density is much higher in a mode less than 1$\mathring{A}$.

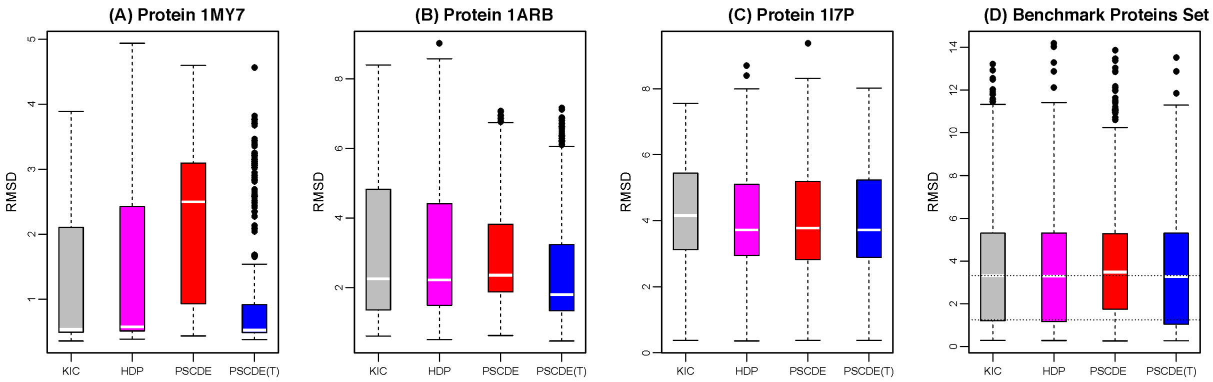


Figure S.9: Side-by-side box-plots for RMSD of KIC with different type of (neighboring) densities. (a-b) RMSD distributions of protein “1MY7” and “1ARB.” Clearly significant improvement occurred when PSCDE(T) has been used with KIC method; (c) RMSD distributions of protein “1I7P.” This is an example that Ramachandran neighboring distributions did not affect the results significantly; (d) RMSD distributions of whole 20 proteins in the benchmark set.
